# Cortical responses before 6 months of life associate with later autism

**DOI:** 10.1111/ejn.13757

**Published:** 2017-11-22

**Authors:** S. Lloyd‐Fox, A. Blasi, G. Pasco, T. Gliga, E. J. H. Jones, D. G. M. Murphy, C. E. Elwell, T. Charman, M. H. Johnson, the BASIS Team, S. Baron‐Cohen, R. Bedford, P. Bolton, H. M. C. Cheung, K. Davies, M. Elsabbagh, J. Fernandes, I. Gammer, J. Guiraud, M. Liew, H. Maris, L. O'Hara, A. Pickles, H. Ribeiro, E. Salomone, L. Tucker, F. Yemane

**Affiliations:** ^1^ Centre for Brain and Cognitive Development Birkbeck, University of London Malet St. WC1E 7HX London UK; ^2^ Department of Psychology University of Cambridge Cambridge UK; ^3^ Department of Psychology Institute of Psychiatry, Psychology, & Neuroscience King's College London London UK; ^4^ Department of Forensic and Neurodevelopmental Science The Sackler Institute for Translational Neurodevelopment Institute of Psychiatry, Psychology, & Neuroscience King's College London London UK; ^5^ Department of Medical Physics and Biomedical Engineering University College London London UK

**Keywords:** ASD, fNIRS, infant, social stimuli, vocalizations

## Abstract

Autism spectrum disorder (ASD) is a common, highly heritable, developmental disorder and later‐born siblings of diagnosed children are at higher risk of developing ASD than the general population. Although the emergence of behavioural symptoms of ASD in toddlerhood is well characterized, far less is known about development during the first months of life of infants at familial risk. In a prospective longitudinal study of infants at familial risk followed to 36 months, we measured functional near‐infrared spectroscopy (fNIRS) brain responses to social videos of people (i.e. peek‐a‐boo) compared to non‐social images (vehicles) and human vocalizations compared to non‐vocal sounds. At 4–6 months, infants who went on to develop ASD at 3 years (*N* = 5) evidenced‐reduced activation to visual social stimuli relative to low‐risk infants (*N* = 16) across inferior frontal (IFG) and posterior temporal (pSTS‐TPJ) regions of the cortex. Furthermore, these infants also showed reduced activation to vocal sounds and enhanced activation to non‐vocal sounds within left lateralized temporal (aMTG‐STG/pSTS‐TPJ) regions compared with low‐risk infants and high‐risk infants who did not develop ASD (*N* = 15). The degree of activation to both the visual and auditory stimuli correlated with parent‐reported ASD symptomology in toddlerhood. These preliminary findings are consistent with later atypical social brain responses seen in children and adults with ASD, and highlight the need for further work interrogating atypical processing in early infancy and how it may relate to later social interaction and communication difficulties characteristic of ASD.

Autism spectrum disorder (ASD) is a common developmental disorder, characterized by impairments in social interaction and communication, the presence of restricted and repetitive behaviours and atypical sensory responses (DSM‐5; American Psychiatric Association, 2013). One of the most challenging features of ASD is its genotypic and phenotypic heterogeneity, which constrains our ability to elucidate the aetiology or developmental pathways that lead to this disorder (Jones *et al*., 2014). It has been suggested that distinct biological pathways may converge at a neural systems level, such as in the ‘social brain’ network (Kaiser *et al*., 2010). It is critical to study ASD early in development to (i) measure the mechanisms underpinning risk vs. resilience in early infancy prior to the onset of atypical behaviours, and (ii) establish intervention strategies and support for families from an earlier age (Green *et al*., 2017; Jones *et al*., 2017). To study early brain development in ASD, attention has recently turned to the study of infants at‐risk of ASD because they are the younger siblings of children with a diagnosis of ASD (Zwaigenbaum *et al*., 2007; Jones *et al*., 2014; Szatmari *et al*., 2016). As ASD is highly heritable, later‐born siblings of diagnosed children are at substantially higher risk of developing ASD or the broader ASD phenotype than the general population (Constantino *et al*., 2010; Ozonoff *et al*., 2011), with one in five of infants with a familial risk of ASD developing ASD themselves (Ozonoff *et al*., 2011). Prospective longitudinal studies of infants at familial risk allow one to search for ‘antecedent biomarkers’ that relate to the onset or severity of later ASD symptoms, and precede their emergence (Johnson *et al*., 2015).

The capacity to engage in complex social interactions is a principle characteristic of what it is to be human, and is thought to be a contributing factor to the development of our culture and civilization (Adolphs, 2009). In typical adults, a network of regions termed the ‘social brain’ (Adolphs, 2009) has been identified, which include the orbitofrontal cortex, inferior frontal gyrus (IFG), amygdala, anterior temporal lobe regions (aMTG‐STG) and posterior superior temporal sulcus – temporoparietal (pSTS‐TPJ) regions. Given the importance of social perception in our day to day interactions, it is noteworthy that converging evidence has implicated atypical brain responses to visual and auditory social stimuli in children and adults diagnosed with ASD (Čeponien≐ *et al*., 2003; Gervais *et al*., 2004; Pelphrey & Carter, 2008; Eyler *et al*., 2012; Zhu *et al*., 2014). Though the developmental causes of this atypical neural phenotype remain unknown, it has been suggested that a problem in one or more of the underlying mechanisms that allow infants to orient towards or to process socially relevant information from early in life may disrupt the typical developmental trajectory of the social brain network (Dawson *et al*., 2005; Johnson *et al*., 2005; Schultz, 2005). As a consequence, atypical neural responses to faces and/or eye contact (Elsabbagh *et al*., 2012) may interfere with the emergence of critical developmental milestones relevant for later social cognitive skills, leading to the well‐established pattern of symptoms of autism becoming embedded and observable by the age of diagnosis (Johnson *et al*., 2005; Dawson *et al*., 2012).

Functional near‐infrared spectroscopy (fNIRS) has been increasingly employed in the study of infants and children due to its ease of use, low cost and capacity for more specific spatial localization compared to electroencephalography (EEG). A key area of research with fNIRS has been in the study of functional specialization to social cues. We use the term ‘social’ in this study in the broadest sense (i.e. that they are visual or auditory human body‐generated stimuli): this does not necessarily imply that these stimuli are intended to be communicative. Through a series of fNIRS studies with infants aged 6 months and under (Grossmann *et al*., 2008, 2013; Lloyd‐Fox *et al*., 2009, 2011, 2014a; Correia *et al*., 2012; Farroni *et al*., 2013), researchers have demonstrated enhanced activation to dynamic visual social stimuli (such as facial eye and mouth movements and nursery rhyme hand actions) in prefrontal, inferior frontal and superior temporal regions of the social brain network. These findings are consistent with patterns of activation found in studies with similar aged infants viewing static social and non‐social stimuli (Otsuka *et al*., 2007; Carlsson *et al*., 2008; Nakato *et al*., 2009) and older infants viewing dynamic social stimuli (Minagawa‐Kawai *et al*., 2009; Ichikawa *et al*., 2010; Urakawa *et al*., 2015; Lloyd‐Fox *et al*., 2017) and fMRI studies with adults (Allison *et al*., 2000; Pelphrey *et al*., 2005a; Lotze *et al*., 2006; Van Overwalle & Baetens, 2009). Further, auditory social responses localized over regions of the middle and superior temporal gyri and sulci (particularly anterior regions) – to vocalizations and auditory communicative cues – have also been demonstrated in fNIRS studies with young infants (Grossmann *et al*., 2010; Minagawa‐Kawai *et al*., 2011; Lloyd‐Fox *et al*., 2012, 2014a,2014b, 2015), replicating and extending fMRI research with adults (Belin *et al*., 2000). Thus, in these infant studies, regions have been identified (i) over IFG and pSTS‐TPJ that show enhanced activation to visual dynamic social stimuli (i.e. actors playing Peek‐a‐boo) relative to both non‐social static images (i.e. cars, trains) and dynamic videos (i.e. machinery, toys turning); and (ii) over aMTG‐STG that respond to auditory human‐generated stimuli (i.e. non‐communicative and communicative vocalizations) to a greater degree than non‐social stimuli (environmental sounds, i.e. rattles, water). Further, recent research suggests that in the first months of life (0–4 months of age), infants exhibit non‐discriminative and/or larger responses to auditory non‐vocal stimuli relative to vocal stimuli (Grossmann *et al*., 2010; Cristia *et al*., 2014; Lloyd‐Fox *et al*., 2017) in bilateral aMTG‐STG and pSTS‐TPJ regions, which then diminishes with age [i.e. absent in a group of 7 month olds and in longitudinal research by approximately 9–13 months of age when studied from 4 months upwards (Grossmann *et al*., 2010; Lloyd‐Fox *et al*., 2017)]. Whilst over the same time period specialization to auditory vocal stimuli (vocal > non‐vocal sounds) in aMTG‐STG increases and becomes more stable over the second half of the first year of life (5 months onwards) and into toddlerhood (Grossmann *et al*., 2010; Lloyd‐Fox *et al*., 2012, 2017). Interestingly, an fMRI study revealed that at 1–4 months of age infants left temporal cortex responded to any communicative sounds, speech or communicative vocalizations (i.e. laughing, disgust) stronger than to non‐communicative vocalizations (i.e. yawning, coughing) and environmental sounds (i.e. water; Shultz *et al*., 2014). Whilst these studies did not directly investigate the same processing (i.e. Shultz *et al*. did not directly investigate responses in other brain regions or non‐vocal selectivity, and Grossman *et al*., Lloyd‐Fox *et al*. did not separate communicative from non‐communicative sounds) taken together these studies support a developmental pathway of specialization to communicative sounds and speech over the first year of life relative to non‐communicative and environmental sounds.

In contrast to this typical pattern of responses to social cues, previous research particularly later in childhood and adulthood has found evidence of atypical cortical responses to social stimuli in individuals with autism. For example, research with older children and adults showed atypical responses to the perception of visual social human actions (Pelphrey & Carter, 2008). Furthermore, evidence in adults suggests that individuals with autism have difficulties with processing vocal stimuli – such as a lack of preference for their mother's voice (Klin, 1991), impairment in the recognition of mental state within a voice (Rutherford *et al*., 2002) and fMRI research in adults with autism failed to identify vocally selective regions (in response to vocalizations and speech sounds compared with environmental sounds) of the aSTS (Gervais *et al*., 2004). Furthermore, atypical functional lateralization to language has been found in children and adults (Kleinhans *et al*., 2008; Redcay & Courchesne, 2008; Anderson *et al*., 2010; Eyler *et al*., 2012). For example, fMRI research with young children later diagnosed with ASD (Eyler *et al*., 2012), aged 1–4 years, found deficient specialization to language in the left hemisphere which became more pronounced in the older aged participants. However, hypo‐responsiveness of the social brain later in life could be a consequence rather than a cause of ASD social and communication atypicalities.

Of interest, several studies have indicated anatomical and connectivity atypicalities in temporal regions, particularly the STS, in children and adults with ASD (Boddaert *et al*., 2004; Zilbovicius *et al*., 2006; Zhu *et al*., 2014). Whether these atypical visual and auditory responses are linked causally to underlying atypical anatomy from this early in life, or whether atypical early function drives anatomical differences has yet to be established. Examining the brain correlates associated with processing social stimuli at an earlier age will help us to understand the developmental trajectory of these atypicalities further and to define infant autism antecedents.

Recently, researchers have begun to use fNIRS in prospective longitudinal studies of ASD (Fox *et al*., 2013; Keehn *et al*., 2013; Lloyd‐Fox *et al*., 2013). We reported reduced fNIRS activation to both visual and auditory social cues (as described above) in 4‐ to 6‐month‐old infants at high risk of developing ASD when compared with age‐matched low‐risk infants (those without a familial risk of ASD; Lloyd‐Fox *et al*., 2013). These infants have now been followed up at 36 months of age, when a clinical diagnosis of ASD could be ascertained. Previous research allowed us to make the following hypotheses. Firstly, we predicted that within IFG and pSTS‐TPJ social brain regions the HR–ASD infants would show reduced responses to the visual social stimuli compared with the non‐social stimuli relative to the low‐risk (LR) infants. Secondly, given previous findings in low‐risk infant cohorts (Lloyd‐Fox *et al*., 2013, 2017) and adults (Gervais *et al*., 2004), we predicted that the aMTG‐STG region would evidence greater vocal selectivity (vocal > non‐vocal responses) in the low‐risk (LR) infants compared with the HR‐ASD infants. Thirdly, given the channels showing non‐vocal selectivity (non‐vocal > vocal) in pSTS‐TPJ regions seen in the high‐risk group in our previous publication (Lloyd‐Fox *et al*., 2013), we predicted that the HR‐ASD infants were driving this response, and that they would therefore show enhanced non‐vocal selectivity relative to the LR and HR‐noASD groups. Finally, evidence of a broader autism phenotype within individuals with a familial risk of ASD led to the hypothesis that the high‐risk infants who did not go on to develop ASD (HR–noASD) would show intermediate responses relative to the HR‐ASD and low‐risk groups.

## Materials and methods

### Participants and clinical characterization

Recruitment, ethical approval (UK National Health Service National Research Ethics Service London REC 08/H0718/76 and 06/MRE02/73) and informed consent, as well as background data on participating families with high‐ and low‐risk infants, were made available for this study through the BASIS network (http://www.basisnetwork.org). All methods and experimental protocols were approved and carried out in accordance with the NHS and Birkbeck, University of London Ethics Committee guidelines and regulations. Informed consent was obtained from the parent/legal guardian for each participant. Data and research materials supporting the results in the article are stored in the BASIS Network Data Repository and requests for data should go through BASIS network data access policies. Families are invited to attend multiple research visits from when their infants are approximately 5 months of age until their children reach 3 years of age or beyond.

Thirty‐six‐four‐ to six‐month‐old infants participated in this study including 20 infant siblings of children with ASD (high risk; 10 female, mean age = 149.35 days, SD = 27.28) and 16 infants who have no family history with ASD (low risk; six female, mean age = 153.81, SD = 25.67). Note that 32 of the 36 infants (16 low risk and 16 high risk) contributed data to a previous publication on low‐ vs. high‐risk infants (Lloyd‐Fox *et al*., 2013). Each high‐risk infant had a full older sibling with a community clinical ASD diagnosis, confirmed using information from the Development and Well‐Being Assessment (DAWBA; Goodman *et al*., 2000) and the Social Communication Questionnaire (SCQ; Rutter *et al*., 2003) by expert clinician (TC). The low‐risk infants were from a volunteer database with no reported family history (first‐degree relative) of ASD and had at least one older full sibling.

During the 36 month visit, a battery of clinical research measures was administered, including the Autism Diagnostic Observation Schedule (ADOS‐2; Lord *et al*., 2012) and the Autism Diagnostic Interview (ADI‐R; Rutter *et al*., 2003); as well as measures of adaptive functioning (Vineland Adaptive Behaviour Scale; Vineland‐II, Sparrow *et al*., 2005), and development (Mullen Scales of Early Learning; MSEL Mullen, 1995). Experienced researchers (TC, GP) reviewed all information and agreed consensus ASD diagnostic outcome according to DSM‐5 (American Psychiatric Association, 2013). Following clinical assessment at 3 years of age, infants were subdivided as follows: (i) HR–ASD: high‐risk infants who went on to receive a later diagnosis of ASD (*N* = 5); (ii) HR–noASD: high‐risk infants who did not go on to develop ASD (*N* = 15); and (iii) LR: low‐risk infants without a familial risk of ASD (*N* = 16). As illustrated in Table 1, at the time point of fNIRS data collection (4–6 months), the three groups of participants were of similar chronological age and developmental ability (according to the MSEL) all within the average range. Furthermore, those infants who were seen but on whom we failed to acquire useable fNIRS data had similar MSEL at 5 months of age (105 (SD 29.1)) and ADOS‐2 scores at 36 months (2.17 (SD 2.16)) to those included in the final data set. When clinical assessments were conducted at 36 months of age (MSEL), the developmental ability of the HR‐ASD group (M = 89.4, SD = 26.5) was lower than both the LR (*t*_18_ = 4.13, *P* = 0.001; LR: M = 122.47, SD = 10.4) and the HR‐noASD group (*t*_18_ = 2.63, *P* = 0.017; HR‐ noASD: M = 112.5, SD = 13.2). The LR group also had a higher score than the HR‐ noASD group (*t*_28_ = 2.291, *P* = 0.03). The recurrence rate of ASD within the high‐risk group (25%) is in line with previous research (Ozonoff *et al*., 2011).

**Table 1:** Participant characteristics

Visit	Measure	LR		HR‐noASD		HR‐ASD	
		Mean (SD)	*N*	Mean (SD)	*N*	Mean (SD)	*N*
4–6 months	Age at visit (months)	5.1 (0.9)	16	4.9 (0.9)	15	4.9 (1.0) *5*	5
	Mullen ELC SS	99.44 (8.5*)*	16	101.4 (12.7)	15	104.75 (3.9) *4*	4
36 months	Age at visit (months)	38.9 (1.5)	15	38.6 (1.7)	15	38.2 (0.5) *5*	5
	Mullen ELC SS	122.47 (10.4)	15	112.53 (13.2)	15	89.4 (26.5) *5*	5
	ADOS‐2 Total CSS	1.60 (1.35)	15	2.0 (1.56)	15	3.80 (3.27) *5*	5
	ADOS‐2 SA CSS	2.33 (1.76)	15	2.0 (1.56)	15	4.20 (3.11) *5*	5
	ADOS‐2 RRB CSS	3.27 (2.22)	15	2.67 (1.84)	15	6.00 (1.41) *5*	5
	ADI – R Social	0.87 (1.2)	15	1.8 (2.6)	15	10.4 (5.4) *5*	5
	ADI – R Comm	0.40 (0.9)	15	2.27 (3.8)	15	13.5 (7.1) *5*	5
	ADI – R BRI	0.07 (0.3)	15	0.2 (0.56)	15	4.80 (2.8) *5*	5
	SRS Total	21.57 (12.2)	14	21.07 (11.3)	15	77.8 (42.8) *5*	5
	Sex (female/male)	6/10		8/7		2/3	

Mullen ELC SS, Mullen Early Learning Composite Standard Score; ADOS‐2, Autism Diagnostic Observation Scale, 2nd edition; CSS, Calibrated Severity Score; SA, Social Affect; RRB, Restricted and Repetitive Behaviours; ADI‐R, Autism Diagnostic Interview‐Revised; Comm, Communication; BRI, Behaviours and Repetitive Interests; SRS, Social Responsiveness Scale.

The fNIRS data presented in this study originate from the first visit that the infants attended as part of the prospective long‐term project at the Centre for Brain and Cognitive Development. A further 24 infants (60% inclusion rate) participated but were excluded from the study (17 high‐risk infants, seven low‐risk infants), details of which are found in the Data Processing section of the Methods. Note that 37 high‐risk and 23 low‐risk infants took part in this study, hence the disparity in numbers between groups.

### Experimental procedures

**Figure 1:**
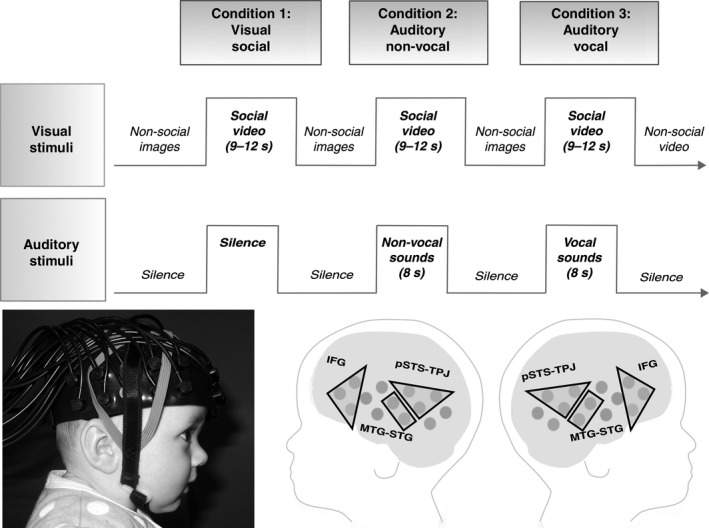
Upper panel: stimulus presentation. Lower panel: An infant wearing the fNIRS headgear (left) and the three ROIs projected on a schematic of the fNIRS measurement channels (right) – inferior frontal gyrus – IFG, posterior portions of the superior temporal sulcus region – temporoparietal junction – pSTS‐TPJ and anterior portions of the middle temporal gyrus – superior temporal gyrus – aMTG‐STG. Parental consent was obtained for the publication of this photo.

Infants wore custom‐built fNIRS headgear (Lloyd‐Fox *et al*., 2010) consisting of two source‐detector arrays (see Fig. 1), containing a total of 26 channels (source‐detector separations; 2 cm) covering frontal and temporal areas. They were tested with the UCL‐NIRS topography system (Everdell *et al*., 2005), which used two continuous wavelengths of source light at 770 and 850 nm. Based on an understanding of light transport, and given that the cortex is approximately 0.5 cm from the skin surface in inferior frontal and temporal regions in this age group (Salamon *et al*., 1990), the channel separation used in this study was estimated to penetrate up to a depth of approximately 1 cm from the skin surface, potentially allowing measurement of both the gyri and parts of the sulci near the surface of the cortex. Before the infants began the study, head measurements were taken to align the headgear with 10–20 coordinates (Lloyd‐Fox *et al*., 2012). Measurements from this group of infants showed that the average head circumference was 43.1 cm (SD 1.63). With the use of the co‐registration MRI‐fNIRS data from our recent work (Lloyd‐Fox *et al*., 2014b), we can approximate the underlying cortical anatomy of the fNIRS channels used in this study. Indeed a third of the infants that contributed data to the generation of the standardized scalp surface map of fNIRS channel coordinates to cortical anatomy (Lloyd‐Fox *et al*., 2014b) are from the present cohort. Therefore, we are confident that we can localize our investigation to specific regions of the social brain network and draw comparisons with findings from adult populations.

Once the fNIRS headgear was placed on their heads, the infants sat on their parent's lap in a dimly lit and sound‐attenuated room. The visual stimuli were displayed on a 117‐cm plasma screen with a viewing distance of approximately 100 cm, and two external speakers were placed just behind this screen to present the auditory stimuli. The parent was instructed to refrain from interacting with the infant during the stimuli presentation unless the infant became fussy or sought their attention. A restriction of studying auditory processing in awake infants of this age is that they need to be presented with concurrent visual stimulation to prolong their attention and reduce infant movement and thus artefact in the signal. We thus chose to combine the presentation of the visual and auditory stimuli to maximize the number of valid trials. Consequently, the auditory stimuli were always in the presence of a trial of visual social stimuli. The experimental condition trials alternated one after the other, with a reference trial (silent presentation of visual static non‐social images) between each. The three types of experimental conditions [visual social (silent) (V‐S), auditory vocal (with visual social) (A‐V), auditory non‐vocal (with visual social) (A‐NV)] were presented in the same order across infants in a repeating complex loop (V‐S, A‐NV, A‐V, V‐S, A‐V, A‐NV, V‐S, A‐V, A‐NV, V‐S, A‐NV, A‐V) of trials (single presentation of a condition) until the infants became bored or fussy as judged by the experimenter who was monitoring their behaviour. The sequence of stimulus presentation is illustrated in Fig. 1.

#### Visual social stimuli

These consisted of full‐colour, life‐size (head and shoulders only) videos displayed for 9–12 s per trial. Each trial consisted of two video clips (4–6 s in length) drawn from a selection of six different videos. These videos were of female adults (two actors) who performed one of three different sequences – either they (i) moved their eyes left or right, or performed hand games – (ii) ‘Peek‐a‐boo’ and (iii) ‘Incy Wincy Spider’. To avoid inducing anticipatory brain activity and control for effects of attention, rather than repeat the same video continuously, we chose to present infants with a selection of video clips over the session. During the reference trials, visual stimuli were displayed, which consisted of full‐colour still images of different types of transport (i.e. cars and helicopters). To maintain infant attention, twelve different images were used. During each reference trial (9–12 s), images were randomly selected from these 12 and presented for a pseudorandom duration (1–3 s) before the next image was displayed. Therefore, infants’ viewed, on average, 4–6 images per reference trial.

#### Auditory stimuli

During the presentation of visual stimuli, the infants were presented with auditory stimuli at the onset of two of every three of the trials (see Fig. 1). The content and duration of the social videos (9–12 s) were not synchronized with the auditory stimuli. For each experimental trial with auditory stimuli, the sounds were presented for 8 s. These trials included four different sounds (of vocal or non‐vocal stimuli) presented for 0.37–2.92 s each, interleaved by periods of silence (of 0.16–0.24 s). The two auditory conditions were equivalent in terms of average sound intensity and duration (*P* > 0.65). Within the vocal condition trials, infants were presented with four communicative and non‐communicative non‐speech adult vocalizations (coughing, yawning, laughing and crying). Within the non‐vocal condition trials, the infants were presented with four naturalistic environmental sounds (that were not human or animal produced, but were likely to be familiar to infants of this age; running water, rattles, squeaky toys, bells). For both the vocal and non‐vocal trials, there were two different combinations of four sounds: these were drawn at random for each experimental trial. Vocal and non‐vocal stimuli were chosen from the Montreal Affective Voices audio collection (for more detail, see Belin *et al*., 2008) and the stimuli of the voice functional localizer (http://vnl.psy.gla.ac.uk/resources_main.php). Additional non‐vocal stimuli (toy sounds) were also recorded by the authors (Blasi *et al*., 2011).

### Data processing and analysis

Within each optical array, light reaching the detectors will have travelled from the sources through the skin, skull and underlying brain tissue. The NIRS system measured changes in the amount of light reaching the detectors, from which the changes in oxy‐haemoglobin (HbO_2_) and deoxyhaemoglobin (HHb) concentration (μMol) were calculated and used as hemodynamic indicators of neural activity (Obrig & Villringer, 2003). The procedure of analysis, using in‐house programs developed in matlab ^®^, followed a similar protocol to previous infant research. Initially, the recorded near infrared attenuation measurements for each infant were analysed, and trials or channels were rejected from further analysis using two criteria: by looking time measures (trials were coded offline by a researcher unfamiliar with the study's aims: > 60% trial looking considered valid) and the quality of the intensity signals (at a channel level), using artefact detection algorithms (Lloyd‐Fox *et al*., 2009, 2010). For each infant, the trials and channels that survived these rejection criteria were entered into further analyses. For each infant, the attenuation signal (from the reflected near‐infrared light) was low‐pass filtered, using a cut off frequency of 1.7 Hz. The data were then divided into blocks consisting of 4 s of the reference trial prior to the onset of the experimental trial, the experimental trial, plus the following reference trial. The attenuation data were detrended with a linear fit between the average of the first and the average of the last 4 s of each block. The data were then converted into changes in concentration (μMol) in HbO_2_ and HHb using the modified Beer Lambert law (Delpy *et al*., 1988) and assuming a differential pathlength factor for infants (5.13; based on Duncan *et al*., 1995). A second level of automatic artefact detection was then conducted on a trial by trial level to identify excessive movement artefacts in the concentration data. Inclusion criteria required each channel to contain valid data in all three experimental conditions. A minimum of three valid trials per condition was set as a threshold for including the data of an infant in the analysis. For a channel to be included in the statistical analysis for a particular infant, at least three valid artefact‐free trials were required. The number of infants that were included for a particular channel was hence variable. Consequently, of the 60 infants tested, 24 were excluded (60% included in sample); 18 – due to an insufficient number of valid trials according to looking time measures, five – due to a high level of rejected fNIRS data (artefact detection algorithms and analyses) and one due to missing the 36‐month clinical assessment as the family moved abroad. Note that while this attrition rate was fairly high – likely a consequence of our 3‐condition design requiring infants’ to view a high number of trials – it did not differ between the low‐ and high‐risk infants and was within the typical range for infant fNIRS studies (Lloyd‐Fox *et al*., 2010; Gervain *et al*., 2011; Cristia *et al*., 2013).

Following this, valid trials for each experimental condition visual social (silent), auditory vocal (social) and auditory non‐vocal (non‐social) were averaged together within channels for each infant, and a time course of the mean concentration change in HbO_2_ and HHb was compiled for each channel. Either a significant increase in HbO_2_, or a significant decrease in HHb, is commonly accepted as an indicator of cortical activation in infant work. If HbO_2_ and HHb were either to increase or decrease significantly in unison, the signal is considered inconsistent with a haemodynamic response to functional activation (Obrig & Villringer, 2003). There was no evidence of regional simultaneous increases/decreases in HbO_2_ and HHb responses in the current data set. While many infant fNIRS studies report significant HbO_2_ responses, far fewer report HHb responses, sometimes through choice, but often because they do not find significant responses (likely due to the magnitude of change in HHb generally being far lower; Lloyd‐Fox *et al*., 2010). In accordance with previous research (Lloyd‐Fox *et al*., 2010; Gervain *et al*., 2011; Cristia *et al*., 2013), we found that the majority of the significant effects were in HbO_2_ and so focused our results on this signal. However, we feel that it is important to report HHb responses to contribute to the field's continued effort to further our understanding of the infant haemodynamic response (see Supporting Information for HHb responses).

Preliminary channel‐by‐channel analyses were conducted for infants within each participant group – following the analysis procedures used in the previous risk group publication (Lloyd‐Fox *et al*., 2013) – to assess the relationship of outcome with the previously reported risk markers of development. For each channel, statistical comparisons (two‐tailed *t*‐tests) were performed on the maximum signal change within the 8–12 and 12–16 s time epochs (post‐experimental stimulus onset) compared to the pre‐stimulus signal average during the non‐social baseline (4‐s epoch immediately before onset of the experimental stimuli). To adjust for multiple comparisons, *P*‐values were displayed both uncorrected (Fig. S1; to follow the method of the previous publication of this low‐ and high‐risk data –Lloyd‐Fox *et al*., 2013) and corrected (Table S1) using a matlab ^®^ False Discovery Rate function (Benjamini & Hochberg, 1995). The specified time windows differ slightly from the window used in the previous publication of our low‐ vs. high‐risk ASD data (Lloyd‐Fox *et al*., 2013) for two reasons. Firstly, due to an analysis error, the window (4–12 s) used in the previous publication was earlier than intended (as explained in an errata – Lloyd‐Fox *et al*., 2016) and so included the initiation of the haemodynamic response rather than being centred over the maximum. For a stimulus trial with this content, and of this length (9–12 s), previous research indicates that one would expect to see the peak beyond, or towards the end of, the condition of interest (i.e. during an 8–16 s window). Secondly, recent research analysing the latency of the response over a large sample with a wide developmental age range from 0 to 2 years has indicated that the use of narrower windows around the peak elicits a more robust and informative marker of developmental specialization of the haemodynamic response to this stimuli (Lloyd‐Fox *et al*., 2017). Therefore, we included two narrower windows in the current analyses: the first (8–12 s) was framed over a window likely to include the maximal responses used in the previous analyses (in Lloyd‐Fox *et al*., 2013), and the second (12–16 s) extended beyond the end of the trial to include the remaining period of maximum activation missed in the previous publication.

Given the uneven sample sizes that are generated by clinical diagnosis of ASD outcome in high‐risk infant cohorts (i.e. approximately 20% go on to develop ASD; Ozonoff *et al*., 2011), group differences were not investigated in the channel‐by‐channel analyses of peak activation. Our main group analyses instead used a linear mixed modelling approach to investigate average responses within ROIs. Furthermore, to provide robust data for later analyses at an individual level, the average time courses for each infant were down‐sampled and baseline‐corrected: by (i) dividing into, and averaging every, 4‐s epoch between 0‐ and 20‐s post‐stimulus onset for each infant; and (ii) subtracting the average response between *t* = 0 to *t* = 0.1 s from each of these epochs. This baseline correction allowed us to remove potential trends remaining from the previous presentation of the reference trial stimuli and focus on interpreting the shape and magnitude of the response to the onset of the experimental condition stimuli.

Based on previous research outlined above, three neuroanatomically defined social brain regions of interest (ROIs) were delineated using the NIRS‐MRI co‐registration scalp anatomical map for 4–7 month olds (Lloyd‐Fox *et al*., 2014b): the regions included inferior frontal gyrus (IFG), anterior middle temporal gyrus/superior temporal gyrus (aMTG‐STG), and posterior superior temporal sulcus/temporoparietal junction (pSTS‐TPJ; see Fig. 1). For the visual contrast, the IFG and pSTS‐TPJ ROIs were used, while for the auditory contrast the aMTG‐STG and pSTS‐TPJ ROIs were selected. For each infant, the ROI hemodynamic responses to each condition visual social (silent), auditory vocal (vocal sounds with visual social), auditory non‐vocal (non‐vocal sounds with visual social) were calculated by averaging responses across the channels within each ROI for each infant. One infant's data (from the HR noASD group) were excluded from the visual condition analyses only as there were no valid data in either of the IFG ROIs. Following this, linear mixed modelling was used to investigate group effects across ROI and hemisphere during the 8–12 and 12–16 s time windows for the visual and auditory contrasts.

## Results

The LR, HR–noASD and HR‐ASD groups did not significantly differ in age, gender, looking behaviour during the task and motion artefact detected in the fNIRS signal (see Tables 1 and 2).

**Table 2:** Behavioural data for the NIRS task at 4–6 months of age

	Low‐risk	HR – no ASD	HR ‐ ASD
Total number of trials presented	13.77 (2.31)	13.8 (2.81)	16.25 (2.87)
Total valid trials	12.54 (2.63)	12.47 (2.39)	12.5 (2.52)
Looking time per trial (%)	94.18 (3.2)	92.59 (5.76)	91.05 (2.4)
Valid trials in Social‐visual condition	4.38 (0.87)	4.13 (0.92)	4.25 (1.26)
Valid trials in Non‐vocal condition	4.08 (1.04)	4.27 (0.8)	4.25 (0.5)
Valid trials in Vocal condition	4.08 (0.95)	4.07 (0.96)	4.0 (0.82)
Excluded channels/condition	1.67 (1.12)	2.04 (2.78)	3.4 (1.79)
Head Circumference (cm)	43.3 (1.81)	42.9 (1.51)	43.2 (1.6)

Note that for each entry the first number refers to the mean value across the group and the bracketed number refers to the SD.

Following the approach used in our previous publication of the risk group responses (Lloyd‐Fox *et al*., 2013), we firstly ran channel‐by‐channel analyses (*t*‐tests) within each outcome group for the two condition contrasts (see Fig. S1 and Table S1). These revealed widespread significant HbO_2_ responses to the visual social stimuli in channels over bilateral IFG and pSTS‐TPJ regions in the LR group (15 channels in inferior frontal and posterior temporal regions). Furthermore, significant responses were also evident in a smaller number of channels within bilateral pSTS‐TPJ regions in the HR‐noASD group (five channels over posterior temporal regions). Channels within these regions were also found to have significant HHb responses (see Table S1). No significant channels were found for the HR‐ASD outcome group. For the auditory contrast, vocal selectivity (vocal > non‐vocal responses) was evident in aMTG‐STG regions for both the LR and HR‐noASD groups (two channels with HbO_2_ responses for each group). Furthermore, non‐vocal selectivity (non‐vocal > vocal HbO_2_ and HHb responses) was evident in pSTS‐TPJ regions for the LR, HR‐ASD and HR‐noASD groups.

Following this, ROI analyses were conducted for the visual and auditory contrasts to assess for differences across the groups (LR, HR‐noASD, HR‐ASD). These focused primarily on the HbO_2_ response, given that this was where the majority of the preliminary channel‐by‐channel significant responses were found.

### The social brain network reveals reduced activation to visual social stimuli in infants who go on to develop ASD

**Figure 2:**
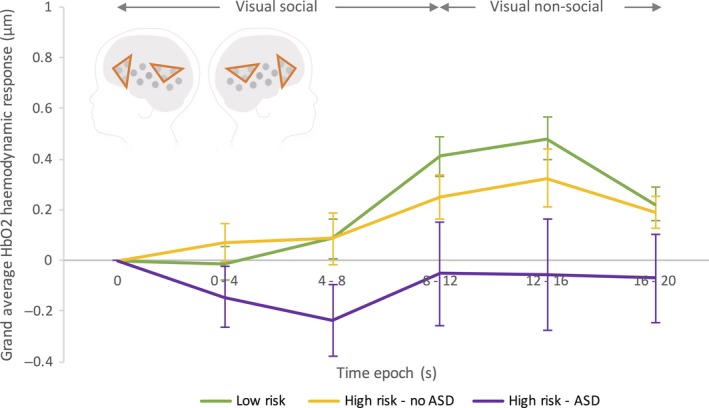
Grand averaged HbO_2_ haemodynamic time courses for the response to the visual social vs. non‐social stimuli (averaged across ROI and Hemisphere) for each of the three participant groups; LR (green), HR‐noASD (yellow), HR‐ASD (purple). Visual social stimuli were presented between 0 to 9–12 s, and visual non‐social stimulus was presented directly before and after this (for 10 s trials). Note the error bars represent standard error. The schematic of the head illustrates the IFG and pSTS‐TPJ ROIs used to generate the time course data.

A linear mixed model analysis of outcome (LR, HR‐noASD, HR‐ASD) × ROI (IFG and pSTS‐TPJ) × hemisphere (left and right) × time epoch (8–12 and 12–16 s) on the average change in HbO_2_ concentration to the visual social stimuli revealed a significant main effect of outcome (*F*_2,32_ = 3.911, *P* = 0.03). Bonferroni‐corrected *post hoc* pairwise comparisons revealed a significantly greater response to the visual social stimuli in the LR outcome group compared with the HR‐ASD group (*P* = 0.026 – LR: M = 0.45, SE = 0.088; HR‐ASD: M = −0.053, SE = 0.16). Responses were also greater in the HR no ASD group (M = 0.28, SE = 0.09) compared with the HR‐ASD group, but the difference was non‐significant (*P* = 0.22). No difference was observed between the LR and HR‐noASD groups (*P* = 0.66). The grand averaged haemodynamic HbO_2_ responses for each group across bilateral IFG and pSTS‐TPJ regions are shown in Fig. 2, and individual responses are shown in Fig. 4.

### Infants who go on to develop ASD show atypical activation to vocal sounds in the left hemisphere

**Figure 3:**
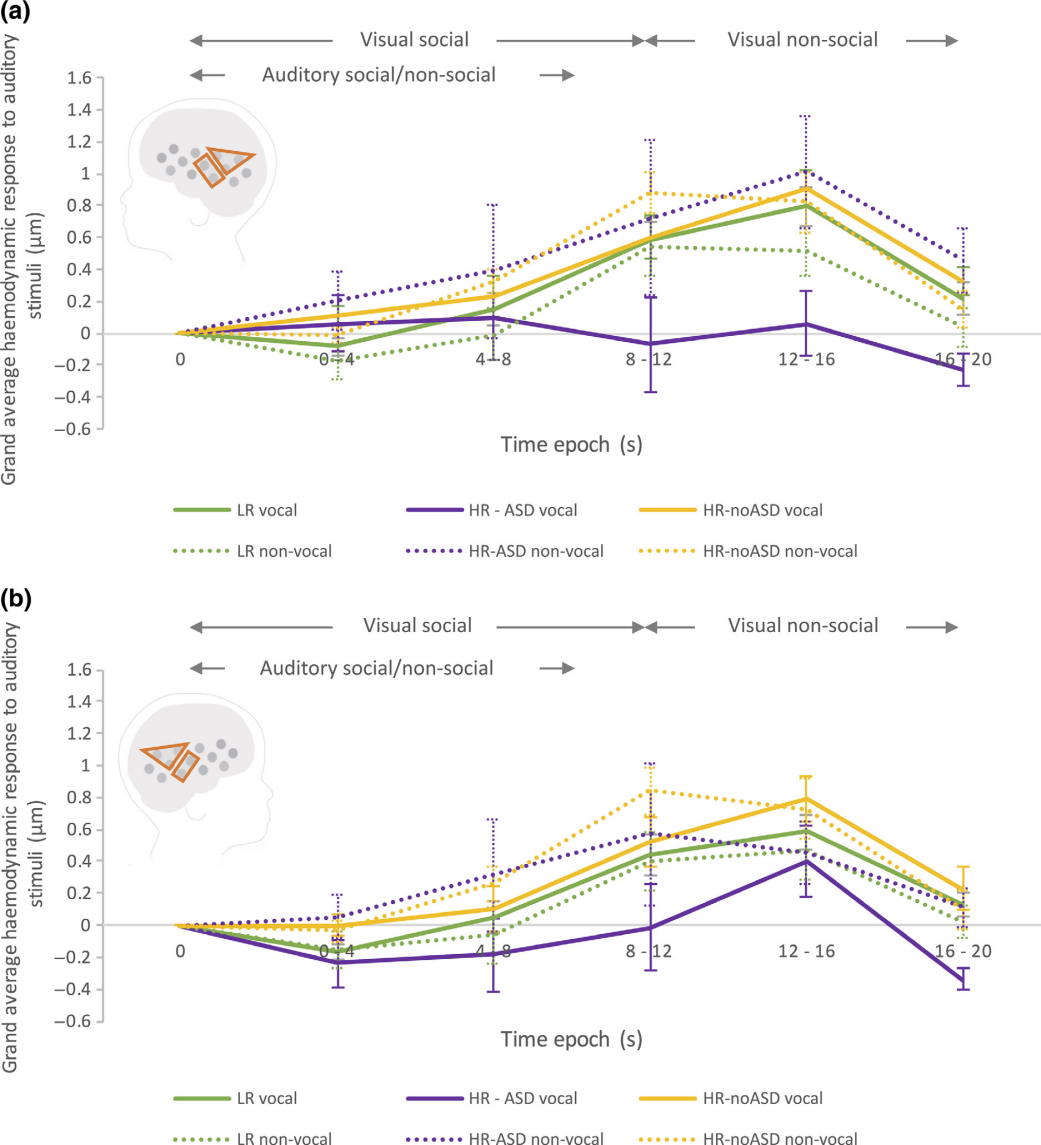
Grand averaged HbO_2_ haemodynamic time courses (across the aMTG‐STG and pSTS‐TPJ ROIs) of the response in the left (a) and right (b) hemisphere to the auditory vocal (solid line) and non‐vocal stimuli (dashed line) for each of the three participant groups; LR (green), HR‐noASD (yellow) and HR–ASD (purple). Auditory stimuli were presented between 0 and 8 s. Note: error bars represent standard error. The schematic of the head illustrates the aMTG‐STG and pSTS‐TPJ ROIs used to generate the time course data.

**Figure 4:**
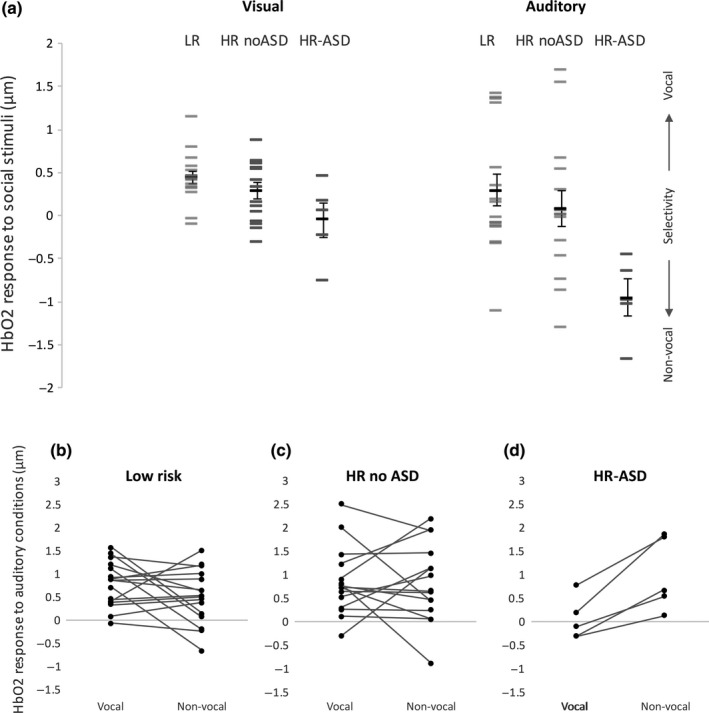
Upper panel (a) – Individual infants’ HbO_2_ responses to the visual (left) and auditory (right) contrasts. Individual visual responses (social relative to non‐social baseline) are averaged across ROI and hemisphere, while auditory responses (vocal minus non‐vocal response) are shown for the left hemisphere only (where the group differences were found). Lower panel – Paired observations of the vocal and non‐vocal responses for the LR (b), HR‐ noASD (c) and HR‐ASD (d) groups.

We considered average HbO_2_ responses in the social brain network ROIs to the auditory vocal vs. non‐vocal stimuli. Difference scores between the responses in time epochs 8–12 and 12–16 s to the auditory vocal and non‐vocal conditions were used in this analysis (i.e. vocal minus non‐vocal response). A linear mixed model analysis of outcome (LR, HR‐noASD, HR‐ASD) × ROI (aMTG‐STG and pSTS‐TPJ) × hemisphere (left and right) × time (8–12 and 12–16 s) revealed a significant main effect of ROI (*F*_1,34.53_ = 5.88, *P *=* *0.021), time (*F*_1,32.36_ = 6.71, *P *=* *0.014) and a marginal, but non‐significant main effect of outcome (*F*_2,33.33_ = 2.83, *P* = 0.073). Furthermore, a significant interaction was found between outcome × hemisphere × time (*F*_2,23.93_ = 5.71, *P *=* *0.009), driven by a differential vocal – non‐vocal response across outcome groups in the left hemisphere (see Fig. 3). To explore the significant interaction with outcome group, Bonferroni‐corrected *post hoc* pairwise comparisons were conducted and revealed a significantly greater vocal > non‐vocal response in the left hemisphere (within time epoch 12–16 s) in the LR outcome group compared with the HR‐ASD group (*P* = 0.008 ‐ LR: M = 0.29, SE = 0.19; HR‐ASD: M = −0.951, SE = 0.33). Furthermore, there was a significantly greater vocal > non‐vocal response in the left hemisphere (within time epoch 12–16 s) in the HR no ASD group (M = 0.09, SE = 0.19) compared with the HR‐ASD group (*P* = 0.033). No significant effects were found for the right hemisphere (*P*s > 0.3). As shown in the grand averaged haemodynamic HbO_2_ responses (Fig. 3), the group differences were driven by vocal selectivity (vocal > non‐vocal) in the LR and HR no ASD groups and non‐vocal selectivity (non‐vocal > vocal) in the HR–ASD group. In particular the HR‐ASD group showed a reduced response to the vocal condition relative to all other vocal and non‐vocal responses across the groups, and individual responses across all 5 HR‐ASD infants (see Fig. 4) showed a higher response to non‐vocal compared with vocal sounds.

### Brain responses at 4–6 months of age associate with ASD symptomology at 3 years

**Figure 5:**
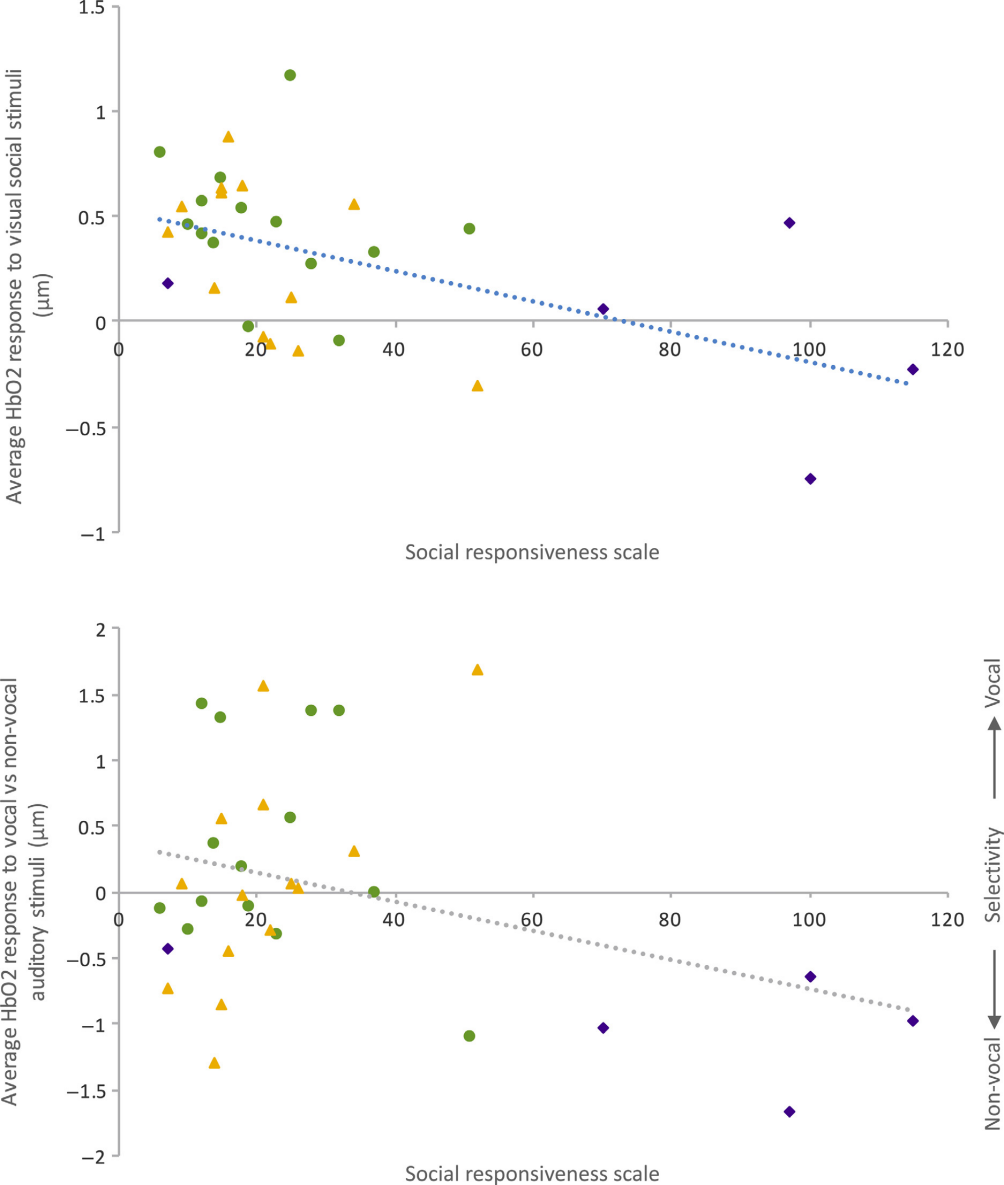
Individual infant's social – non‐social HbO_2_ hemodynamic responses to the visual social stimuli averaged across ROI and hemisphere (left panel) and auditory vocal stimuli for the left hemisphere averaged across ROI (right panel) compared with their Social Responsiveness Scale (SRS) score. LR infants – green circle; HR‐noASD infants – yellow triangle; HR‐ASD infants – purple diamond.

A dimensional approach (relations of quantitative scores in neuroimaging and behavioural measures) to characterizing psychopathology has been advocated (Insel *et al*., 2010; Casey *et al*., 2014) with the aim of better revealing underlying mechanisms. To align with these recommendations, we also investigated the association between brain measures and continuous measures of social abilities across all infants (high and low risk). For this analysis, we used the total score from the Social Responsiveness Scale (SRS) as it captures ASD phenotypic trait variation across both the clinical and subclinical typical range (Constantino, 2012). We investigated the association between the SRS scores and the brain responses associated with significant between‐group effects in our previous analyses as follows: (i) the HbO_2_ hemodynamic response to the visual social stimuli (averaged across ROI, time and hemisphere), and (ii) the HbO_2_ hemodynamic response in the left hemisphere (averaged across ROI within the 12–16 s epoch) to the auditory vocal – non‐vocal contrast. As shown in Fig. 5, a significant negative correlation was found between the SRS score at 36 months and the amplitude of the visual social response (Pearson *r*_32_ = −0.513, *P *=* *0.002), and the auditory vocal – non‐vocal response (Pearson *r*_33_ = −0.349, *P *=* *0.047). This suggests that the lower the HbO_2_ hemodynamic change was to the visual social stimuli and the auditory vocal (compared with non‐vocal) stimuli at 4–6 months of age, the higher the level of ASD behaviours that were evident at 36 months of age across participants. To further investigate whether these findings reflect a population level effect [driven by traits (characteristics) evident across individuals] or were driven by an effect of HR‐ASD outcome we ran a *post hoc* partial correlation analysis, entering outcome group as a covariate. This reduced the significance of the correlation between SRS score and visual social response to a non‐significant trend (*r*_30_ = −0.326, *n* = 32, *P *=* *0.07), indicating that outcome group did have some effect, but was not solely influencing the relationship between the visual social response and ASD behaviours (given that the analysis controlling for group differences still accounts for some of the variance). In contrast, there was no association between SRS score and the vocal response, once outcome group had been factored in (*r*_30_ = −0.04, *n* = 33, *P *=* *0.8), suggesting that outcome group was having a significant influence on the trend seen between the vocal/non‐vocal responses and ASD behaviours.

## Discussion

While we acknowledge that the present study is exploratory, and we are currently following up with increased sample size, to our knowledge, this is the first research to show that functional brain responses before the first 6 months of life are associated with later ASD outcome in toddlerhood. In accord with our hypotheses, our analyses revealed three main findings: (i) Regions of the social brain network (IFG and pSTS‐TPJ) show reduced activation to visual social stimuli in those infants who go on to a later diagnosis of ASD; (ii) Regions of the aMTG‐STG and pSTS‐TPJ in the left hemisphere show reduced activation to vocal relative to non‐vocal sounds in those infants who go on to a later diagnosis of ASD relative to LR and HR‐noASD infants; (iii) Brain responses to these visual and auditory social stimuli at 4–6 months of age associate with parent‐reported symptoms of ASD at 3 years. In contrast, at a behavioural level, previous research of social attention in familial ASD‐risk infants has revealed relatively typical patterns of social orienting at 6 months of age [Ozonoff *et al*. (2010) and Elsabbagh *et al*.(2013); but see Chawarska *et al*. (2013) and Jones & Klin (2013)]. Therefore, atypical cortical responses could be present prior to behavioural manifestations of the disorder, as suggested by recent research with 6 month olds (Emerson *et al*., 2017).

The current analyses were unable to entirely dissociate the responses of the HR–ASD infants from the other participants as significant differences between the HR–ASD and HR–noASD groups were found for the auditory, but not the visual contrast (where only a marginal effect was evident). Importantly, this is a pattern commonly reported in other infant‐risk studies (Elsabbagh *et al*., 2013), with unaffected siblings of children with autism sharing common patterns of atypical activation (‘trait activity’). This has been suggested to reflect a broader autism phenotype seen in other family members (Bolton *et al*., 1994; Piven *et al*., 1997; Ozonoff *et al*., 2014). It is now widely accepted there are multiple contributors to emerging developmental pathology, acting in an additive or multiplicative manner (Bedford *et al*., 2014). Therefore, it is important in future research to establish whether individuals who evidence atypical responses early on and subsequently do not develop ASD may develop compensatory behavioural strategies and/or networks of brain functional responses.

In line with findings in adults (Pelphrey *et al*., 2005b, 2011; Pelphrey & Carter, 2008), we have found that infants who later go on to develop ASD in childhood show a less specialized profile of activation in the social brain network during the perception of complex and dynamic visual social stimuli relative to age‐matched controls. In typically developing neonates, the magnitude of the response to social stimulus in social brain regions correlated with their age in hours over the first few days post‐partum (Farroni *et al*., 2013). It is possible that the typical trajectory of experience‐dependent specialization to socially relevant stimuli that occurs over the first days and weeks of life is altered in those infants who go on to develop ASD. Taken together with other recent findings in prospective infant‐risk studies (Elsabbagh *et al*., 2012; Jones *et al*., 2016a), one possible explanation may be that the rapid temporal and high fidelity processing required by dynamic stimuli is affected in infants who go on to a later diagnosis. A characteristic of interactions with other humans is that it is dynamic and probabilistic, and it may be these features that lead to greater deficits in social perception and cognition. Difficulties with processing these stimuli could lead to lesser engagement with social interaction which would, in turn, delay or detract cortical specialization. In the current study, the reduced responses to visual social stimuli were also found to be associated with increased traits of atypical social responsiveness (characteristics linked to ASD) across all infants. Interestingly, reduced activation to socially relevant visual and tactile information in adults has also been associated with the degree of traits of ASD found within individuals (Kaiser *et al*., 2010; Voos *et al*., 2013). Furthermore, heritable alterations in brain responses to social attention in low‐risk 6‐month‐old infants have been linked to an infant endophenotype of social motivation, associated with ASD, in primary caregiver biological parents (Jones *et al*., 2016b).

The atypical cortical processing of auditory vocal sounds in left aMTG‐STG and pSTS‐TPJ regions in the HR‐ASD infants, closely resembles findings from older children and adults with ASD (Boddaert *et al*., 2003; Gervais *et al*., 2004; Eyler *et al*., 2012), showing atypical vocal selectivity in MTG‐STG regions and atypical left temporal specialization for language in 1–4 year olds with ASD. In typical development, research has shown a decrease in the degree of activation to non‐vocal auditory stimuli over the first 6 months of life (Grossmann *et al*., 2010; Lloyd‐Fox *et al*., 2012, 2017; Cristia *et al*., 2014; Shultz *et al*., 2014). These previous findings link together to show an age‐related shift of auditory specialization to communicative sounds and speech over the first year of life. It is possible that specialization to communicative sounds is developmentally delayed in infants who go on to develop ASD, thus the immature pattern of selectivity evident here. Importantly, it is the lack of a vocal haemodynamic response in left aMTG‐STG and pSTS‐TPJ regions, that is driving the non‐vocal selectivity in the HR‐ASD infants, as the non‐vocal haemodynamic time courses in the HR‐ASD infants are similar to the non‐vocal and vocal responses in the other two groups (LR and HR‐noASD). Therefore, these HR‐ASD infant responses in auditory areas are stimulus‐dependent and not associated with a general reduction in cortical activation or putative atypical vascular responses. A further possibility is that HR‐ASD infants at this age show a delayed response to vocalizations, which over time become equivalent to their response to non‐vocal sounds, as reflected by the eventual lack of vocal and non‐vocal selectivity found in adults with ASD (Gervais *et al*., 2004). We would thus predict a reduction in non‐vocal selectivity to occur at a slower rate relative to the trajectory outlined earlier for low‐risk individuals.

It is important to acknowledge a number of factors which limit the strength of our interpretation of the current results. Firstly, the number of individuals who went on to develop ASD are small. Given the phenotypic heterogeneity and genetic variability seen across individuals with ASD, we should be careful not to infer any predictive qualities of these results at this stage. The dimensional relationship between ASD symptomology and the social visual and auditory responses across the whole sample are in support of our group level findings. Therefore, it will be important to see how the pattern of atypical responses emerge and interplay in larger fNIRS samples and whether they support other recent predictive findings in functional neuroimaging research in ASD infant cohort studies (Emerson *et al*., 2017).

Secondly, we wish to address several caveats of the stimuli used in the current study. Regarding the visual responses, we acknowledge that the degree of motion differed across conditions. However, previous research with typically developing infants has found no evidence of significant effects to non‐social dynamic stimuli in the ROIs under investigation in the current study (Lloyd‐Fox *et al*., 2009; Farroni *et al*., 2013). Furthermore, a recent high‐risk infant ASD study, which is based on a visual dynamic social – dynamic non‐social contrast, replicated our previous risk group differences (Lloyd‐Fox *et al*., 2013) confirming that the social visual low‐risk > high‐risk group effects are not driven by the dynamic nature of the stimuli alone (Braukmann *et al*., 2018). With regard to the auditory effects, as we acknowledged previously (Lloyd‐Fox *et al*., 2013), there is a possibility that infants at‐risk of ASD process multisensory stimuli differently from their typically developing counterparts, due to the incongruent presentation of visual and auditory stimuli. Though we were careful to ensure the visual and auditory stimuli were non‐synchronous and pseudo‐randomized, our design was clearly restricted by what is possible with infants in a limited time period. We do not believe that cross‐modal effects are a significant contributor as the low‐risk findings largely replicate those of previous fMRI and fNIRS studies in adults and infants, whether or not the auditory stimuli were accompanied by visual stimuli (Belin *et al*., 2000; Grossmann *et al*., 2010; Blasi *et al*., 2011; Lloyd‐Fox *et al*., 2012). Indeed the multimodal presentation in Grossmann *et al*. (2010) used non‐human dynamic visual stimuli alongside the vocal and non‐vocal auditory stimuli yet still found similar patterns of voice‐selective activation to the current study using social dynamic visual stimuli. However, given recent evidence suggesting that individuals with ASD may have atypical multisensory processing, for example the presence of speech has been shown to disrupt processing of videos of complex social stimuli (Shic *et al*., 2014), in future work, we aim to disentangle how different components of the current stimuli may have contributed to the atypical response seen here.

Thirdly, whilst previous research with adults (i.e. Belin *et al*., 2000; Pelphrey *et al*., 2005a) using similar stimuli has not identified other regions of activation, we cannot be certain whether the HR‐ASD infants were responding less to the social stimuli in the current study, or whether they instead employed an atypical network of brain regions which were not measured by our channel configuration. Future work with a more extensive array of fNIRS channels or functional MRI (for the auditory contrasts) may help elucidate this further.

For example, while we focused on HbO_2_ responses in the current work, our preliminary channel‐by‐channel analyses suggests potential differences in vascular coupling between HbO_2_ and HHb across groups (e.g. with HR‐noASD infants showing widespread HHb responses to the auditory stimuli in contrast to isolated regions of significance in the HbO signal: see Table S1). Future research should therefore interrogate the specifics of the neurovascular response using a mathematical model of cerebral blood flow and metabolism (Banaji *et al*., 2008) to more fully interpret individual differences in stimulus‐driven hemodynamic responses.

We have identified the earliest departure from typical development of brain function in individuals who later receive a diagnosis of ASD reported to date. Given that the development of ASD in infancy is dynamic and cognitive brain and behaviour markers of risk may change with age (Jones & Klin, 2013; Jones *et al*., 2016a), it is important to follow‐up these findings to extend the research across a wider age range and multiple underlying core constructs (Jones *et al*., 2014), to interrogate early biomarkers in greater depth. Future research should further explore whether there is a relationship with the complexity and multisensory nature of the stimuli used, how these responses relate to genetic and other neural mechanisms, and how they may in turn link to more complex social learning and social interaction throughout later development.

## Conflict of interest

We declare that the authors have no competing interests, or other interests that might be perceived to influence the results and/or discussion reported in this paper.

## Author contributions

S.L‐F. contributed to all aspects of this experiment: design, data collection and data analyses. A.B. & G.P. contributed to the design and data collection. C.E.E., T.C., D.M & M.H.J. contributed to the design. The BASIS team contributed to recruiting and implementing the protocol for the BASIS Phase 2 cohort. All authors (S.L‐F., A.B., G.P., T.G., E.J.J., C.E.E., T.C., D.G.M. & M.H.J.) contributed to interpreting the results and to writing the manuscript.

## Data accessibility

Data are accessible through the British Autism Study of Infant Siblings (BASIS) Network, details of which are available at http://www.basisnetwork.org.

**Abbreviations**
ADI‐R: Autism Diagnostic Interview
ADOS‐2: Autism Diagnostic Observation Schedule
ASD: Autism Spectrum Disorders
BASIS: British Autism Study of Infant Siblings
DAWBA: Development and Well‐Being Assessment
EEG: Electroencephalography
fMRI: functional magnetic resonance imaging
fNIRS: functional near‐infrared spectroscopy
HbO_2_: oxy‐haemoglobin
HHb: deoxyhaemoglobin
HR‐ASD: high‐risk ASD group
HR‐noASD: high‐risk noASD group
IFG: inferior frontal gyrus
LR: low‐risk group
MSEL: Mullen Scales of Early Learning
MTG‐STG: middle temporal gyrus – superior temporal gyrus region
pSTS‐ TPJ: posterior superior temporal sulcus‐temporoparietal junction region
ROI: region of interest
SCQ: Social Communication Questionnaire
SRS: Social Responsiveness Scale

## Supporting information

Fig. S1. A schematic of the infant head showing channels with statistically significant HbO_2_ responses for the low risk (green), high risk – no ASD (yellow) and high risk – ASD (purple) groups in the channel‐by‐channel analysis for the visual social vs. baseline, auditory vocal > non‐vocal and non‐vocal > vocal contrasts (*t*‐test, two‐tailed, *P* < 0.05).Table S1. Channel by channel significant increases in HbO_2_/decreases in HHb concentration during the Visual and Auditory contrasts for the LR, HR – noASD and HR – ASD participants.

## Affiliations for BASIS Consortium

The BASIS team in alphabetical order: S. Baron‐Cohen (Autism Research Centre, University of Cambridge, UK); R. Bedford (NIHR Biomedical Research Centre, Kings College London, UK); P. Bolton (NIHR Biomedical Research Centre, Kings College London, UK); H. M. C. Cheung (Centre for Brain and Cognitive Development, Birkbeck, University of London, London, UK); K. Davies (Centre for Brain and Cognitive Development, Birkbeck, University of London, London, UK); M. Elsabbagh (Department of Medicine, McGill University); J. Fernandes (Centre for Brain and Cognitive Development, Birkbeck, University of London, London, UK); I. Gammer (NIHR Biomedical Research Centre, Kings College London, UK); J. Guiraud (Centre for Brain and Cognitive Development, Birkbeck, University of London, London, UK); M. Liew (Centre for Brain and Cognitive Development, Birkbeck, University of London, London, UK); H. Maris (Centre for Brain and Cognitive Development, Birkbeck, University of London, London, UK); L. O'Hara (Centre for Brain and Cognitive Development, Birkbeck, University of London, London, UK); A. Pickles (NIHR Biomedical Research Centre, Kings College London, UK); H. Ribeiro (Centre for Brain and Cognitive Development, Birkbeck, University of London, London, UK); E. Salomone (Università degli Studi di Torino); L. Tucker (Centre for Brain and Cognitive Development, Birkbeck, University of London, London, UK); F. Yemane (Centre for Brain and Cognitive Development, Birkbeck, University of London, London, UK).
