# Supplementary material for: Cortical responses before 6 months of life associate with later autism
Source: Eur J Neurosci. 2017 Nov 22;47(6):736–49. doi: 10.1111/ejn.13757 (PMC5900943; doi:10.1111/ejn.13757)
Supplement: Supplementary file 1 — Fig. S1. A schematic of the infant head showing channels with statistically significant HbO2 responses for the low risk (green), high risk – no ASD (yellow) and high risk – ASD (purple) groups in the channel‐by‐channel analysis for the visual social vs. baseline, auditory vocal > non‐vocal and non‐vocal > vocal contrasts (t‐test, two‐tailed, P < 0.05).Table S1. Channel by channel significant increases in HbO2/decreases in HHb concentration during the Visual and Auditory contrasts for the LR, HR – noASD and HR – ASD participants. [file EJN-47-736-s001.docx]

**Supplementary Information**

**Supplementary Figure S1:** A schematic of the infant head showing channels with statistically significant HbO_2_ responses for the low risk (green), high risk – no ASD (yellow) and high risk – ASD (purple) groups in the channel-by-channel analysis for the *visual social versus baseline, auditory vocal > non-vocal and non-vocal > vocal contrasts* (t-test, two-tailed, p < 0.05). Peak responses were analysed during the 8-12 and 12-16s time epochs following stimulus presentation (see Supplementary Table S1 for full details of results).

| **Low risk** | | | | | | | | | | | | | | | | **HR - noASD** | | | | | | | | | | | | | | | | | | | |
| --- | --- | --- | --- | --- | --- | --- | --- | --- | --- | --- | --- | --- | --- | --- | --- | --- | --- | --- | --- | --- | --- | --- | --- | --- | --- | --- | --- | --- | --- | --- | --- | --- | --- | --- | --- |
|  |  | |  | | | | | | | | | | | | |  | | |  | |  | | | | | | | | | | | | | | |
|  | **Visual Social > Non-Social (HbO_2_)** | | | | | |  | **Auditory Vocal > Non-Vocal (HbO_2_)** | | | | | | | |  | | | **Visual Social > Non-Social (HbO_2_)** | | | | | | | | |  | **Auditory Vocal > Non-Vocal (HbO_2_)** | | | | | | |
| **Ch** | | *TW* | | *t* | *p* | *N* | **Ch** | *TW* | | *t* | *p* | | | | *N* | **Ch** | | | | *TW* | | *t* | | *p* | | | *N* | **Ch** | | *TW* | *t* | *p* | | | *N* |
| **1** | | 8-12s | | 2.4 | 0.031 | *15* | **29** | 8-12s | | 2.91 | 0.012 | | | | *15* | **18** | | 8-12s | | | | 2.62 | | 0.024 | | | *12* | **6** | | 12-16s | 2.6 | 0.022 | | | *14* |
| **2** | | 12-16s | | 2.48 | 0.025 | *16* | **28** | 12-16s | | 2.17 | 0.046 | | | | *16* | **18** | | 12-16s | | | | 3.63 | | 0.004 | | | *12* | **9** | | 12-16s | 2.22 | 0.044 | | | *14* |
| **5** | | 12-16s | | 2.74 | 0.017 | *14* |  | **(HHb)** | | | | | | | | **25** | | 8-12s | | | | 2.31 | | 0.038 | | | *14* |  | |  |  |  | | |  |
| **10*** | | 12-16s | | 3.78 | 0.002 | *14* | **21** | 12-16s | | 2.33 | 0.04 | | | 16 | | **34** | | 8-12s | | | | 3.37 | | 0.005 | | | *15* |  | | **Auditory Non-Vocal > Vocal (HbO2)** | | | | | |
| **14*** | | 8-12s | | 3.7 | 0.002 | *15* |  |  | |  |  | | | |  | **36** | | 12-16s | | | | 2.51 | | 0.026 | | | *14* |  | |  |  |  |  |  |  |
| **14*** | | 12-16s | | 4.52 | <0.001 | *15* |  | **Auditory Non-Vocal > Vocal (HHb)** | | | | | | | | **37** | | 8-12s | | | | 2.23 | | 0.044 | | | *14* | **Ch** | | *TW* | *t* | *p* | | | *N* |
| **15*** | | 8-12s | | 3.48 | 0.004 | *15* |  |  |  |  |  |  |  |  |  | **37*** | | 12-16s | | | | 4.36 | | <0.001 | | | *14* | **15** | | 8-12s | 2.29 | 0.04 | | | 14 |
| **15*** | | 12-16s | | 6.29 | <0.001 | *15* | **Ch** | *TW* | *t* | | | *p* | *N* | | |  | | **(HHb)** | | | | | | | | | | **23** | | 8-12s | 2.25 | 0.042 | | | 14 |
| **18** | | 8-12s | | 2.46 | 0.03 | *13* | **13** | 8-12s | | 2.71 | 0.016 | | | | 16 | **10** | | 12-16s | | | | | 3.66 | | 0.003 | *15* | | **34** | | 8-12s | 2.34 | 0.036 | | | 14 |
| **18*** | | 12-16s | | 3.7 | 0.003 | *13* | **13** | 12-16s | | 2.55 | 0.022 | | | | 16 |  | |  | | | |  | |  | | |  |  | | **(HHb)** | | | | | |
| **21** | | 8-12s | | 2.17 | 0.046 | *16* | **15** | 2.42 | | 2.42 | 0.03 | | | | 15 |  | |  | | | |  | |  | | |  | **13** | | 8-12s | 3.18 | | 0.007 | 15 | |
| **23*** | | 12-16s | | 3.14 | 0.007 | *16* |  |  | |  |  | | | |  |  | |  | | | |  | |  | | |  | **14** | | 8-12s | 2.75 | 0.017 | | | 14 |
| **32** | | 12-16s | | 2.18 | 0.046 | *16* |  |  | | | | | | | |  | |  | | | |  | |  | | |  | **17** | | 8-12s | 2.66 | 0.022 | | | 13 |
| **33** | | 8-12s | | 2.59 | 0.021 | *15* |  |  |  |  |  |  |  |  |  |  | |  | | | |  | |  | | |  | **25** | | 8-12s | 2.8 | 0.015 | | | 14 |
| **33** | | 12-16s | | 2.79 | 0.014 | *15* |  |  |  | | |  |  | | |  | |  | | | |  | |  | | |  | **32** | | 8-12s | 2.4 | 0.03 | | | 15 |
| **34*** | | 8-12s | | 5.52 | <0.001 | *16* |  |  |  | | |  |  | | |  | |  | | | |  | |  | | |  | **33** | | 8-12s | 2.34 | 0.035 | | | 15 |
| **34** | | 12-16s | | 4.03 | 0.001 | *16* |  |  | |  |  | | | |  | **HR - ASD** | | | | | | | | | | | | | | | | | | | |
| **36** | | 12-16s | | 2.62 | 0.02 | *15* |  |  | |  |  | | | |  |  | | | | | | | | | | | | **Auditory Non-Vocal > Vocal (HbO_2_)** | | | | | | | |
| **37** | | 12-16s | | 6.07 | <0.001 | *16* |  |  | |  |  | | | |  |  | |  | | | |  | |  | | |  | **Ch** | | *TW* | *t* | *p* | | | *N* |
| **(HHb)** | | | | | | |  |  | |  |  | | | |  |  | |  | | | |  | |  | | |  | **15** | | 12-16s | 3.21 | 0.049 | | | 4 |
| **Ch** | | *TW* | | *t* | *p* | *N* |  |  | |  |  | | | |  |  | |  | | | |  | |  | | |  |  | |  |  |  | | |  |
| **4** | | 12-16s | | 2.77 | 0.014 | 16 |  |  | |  |  | | | |  |  | |  | | | |  | |  | | |  |  | |  |  |  | | |  |
| **15** | | 8-12s | | 2.76 | 0.015 | 15 |  |  | |  |  | | | |  |  | |  | | | |  | |  | | |  |  | |  |  |  | | |  |
| **15** | | 12-16s | | 3.88 | 0.001 | 15 |  |  | |  |  | | | |  |  | |  | | | |  | |  | | |  |  | |  |  |  | | |  |
|  |  | |  | | | | | | | | | | | | |  |  | | | |  | | | | | | | | | | | | | | |

**Table S1:** Channel by channel significant increases in HbO_2_ / decreases in HHb concentration during the Visual and Auditory contrasts for the LR, HR – noASD and HR – ASD participants.
