# Supplementary material for: Cortical responses before 6 months of life associate with later autism
Source: Eur J Neurosci. 2017 Nov 22;47(6):736–49. doi: 10.1111/ejn.13757 (PMC5900943; doi:10.1111/ejn.13757)
Supplement: Supplementary file 2 [file EJN-47-736-s002.pdf]

EJN European Journal of Neuroscience FENS Federation of European Neuroscience Societies

Cortical responses before six months of life associate with later autism

Lloyd-Fox, S., Blasi, A., Pasco, G., Gliga, T., Jones, E.J., Murphy, D. G., Elwell, C.E., Charman, T. & Johnson, M.H.

Review timeline:

Submission date:	21 December 2016
Editorial Decision:	02 April 2017
Revision received:	26 June 2017
Editorial Decision:	29 August 2017
Revision received:	13 September 2017
Editorial Decision:	28 September 2017
Revision received:	05 October 2017
Accepted:	17 October 2017

Editor: Sophie Molholm

1st Editorial Decision 02 April 2017

Dear Dr. Lloyd-Fox,

We have received two reviews of your manuscript from experts in the field. Reviewer 2 stresses the value of looking for diagnostic markers of autism in at risk infants. However it is also rightly noted by both reviewers that the small number of confirmed cases at follow-up (5) makes it impossible to draw strong conclusions from these data. As such the findings can only be presented as exploratory in nature, and the title and abstract are misleading. A number of additional concerns are raised by the reviewers that will need to be addressed, including shortening the discussion, not drawing conclusions based on non-significant results, and being more balanced (and in some cases more detailed) in consideration of the relevant literature. We also ask that you consider our editorial on presentation of data in figures and modify your figures accordingly (http://onlinelibrary.wiley.com/doi/10.1111/ejn.13400/abstract).

Obviously, a resubmission of your manuscript could only be considered on the basis of extensive revisions. When you prepare your re-submission, please consult the Author's Checklist provided at the bottom on this email, notably with regard to characterization of reagents and reporting of statistical analyzes in the Results section and Figure legends.

Thank you for submitting your work to EJN.

Kind regards,

John Foxe & Paul Bolam
co-Editors in Chief, EJN

Reviews:

Reviewer: 1 (Sasha Key, Vanderbilt Kennedy Center, USA)

Comments to the Author
This is a retrospective analysis of infant fNIRS data with subjects grouped based on the 36-month diagnostic classification. The authors propose reduced cortical activity in response to visual and auditory social stimuli in infants 4-6 months of age as the plausible neural signature of later ASD diagnosis.

The authors are clearly aware of their study limitations (and did an excellent job in the discussion addressing them), but the title conveys a stronger message than is warranted by the preliminary nature of the findings. Similarly, trend-level results (e.g., for the visual social-nonsocial analysis) may not be sufficient to support the argument for an endophenotype.

Given the previously published work from this group using the same paradigms, it is surprising that the

analyses are only retrospective (why not explore the prospective associations between the reported fNIRS measures indexing group differences at 4-6 months and the later outcomes?), and the hypotheses are not specific in regard to the ROIs.

Of particular concern is that the fNIRS findings of this retrospective analysis do not appear to align well with the channels/ROIs previously identified in infant data as sensitive to risk group membership. Furthermore, in this manuscript, the low-risk and HR-noASD groups demonstrated no evidence of vocal-nonvocal sound discrimination (the activation difference scores near zero), which contradicts previous reports of greater sensitivity to vocal than nonvocal stimuli in 4-6-month-old LR infants.
The discussion does not address these discrepancies, which brings into question the relative utility of the results from this and/or prior studies.

Additional comments:
The introduction could be strengthened by providing a more detailed background (e.g., p.4 “we have identified regions…” – which ones? P.5 “we reported diminished fNIRS activation…” – in what specific regions?). Similarly, please clarify the typical time course of emerging speech selectivity (p.21 “a few months later” is to generic).

Visual and auditory hemodynamic data are presented inconsistently: only difference values are presented in Fig.2 (visual) while separate conditions are plotted in Fig.3 (auditory).

Please clarify the rationale for pairing the auditory stimuli with social videos and not a different visual stimulus (bubbles? abstract screensavers?).

Reviewer: 2 (Heather Bortfeld, University of California- Merced, USA)

Comments to the Author
EJN-2016-12-24223 (ASD): Cortical signature of autism evidence before six months of life

The manuscript reports behavioral and neurophysiological data collected from ~36 children at two times points: first, between 4 and 6 months and again at 36 months. Children were either low risk or high risk for Autism Spectrum Disorder. High risk children were infant siblings of children who had been clinically diagnosed as having ASD. The authors compare neural responses collected at Time 1 using functional near infrared spectroscopy in response to social/nonsocial visual/auditory combinations of stimuli based on the diagnostic status of the children at Time 2. They interpret their findings as indicating that fNIRS can be used to identify a neural marker of ASD within the first year of life.

I have a mixed assessment of this work. On the one hand, it is important to identify early markers of ASD for targeting early intervention. On the other, these results are centered on only five children who ultimately were diagnosed with ASD. Making any claims based on an N of 5 would make me uncomfortable; making claims about a “cortical signature of autism” as the title and content of this manuscript do make me doubly so.

The authors do a good job justifying every aspect of the data that doesn’t cohere and they anticipate a wide variety of counter arguments in their discussion section (so much so, it seems safe to say that this work has been rejected in various ways by various journals). The problem is that their conclusion is an easy one given the huge body of data preceding them that would point to the possibility of a neural signature for ASD. The problem with their data is the relatively gross localization of their probe (no digital localization was conducted) and the averaging across large chunks of cortex their data are based on. The brain regions in question here are relevant to all sorts of processing. If one didn’t have the prior knowledge of what the ASD data should show that would follow a clear story, the data could very well be accounted for in a variety of other ways. The primary alternative explanation I kept returning to was that whatever effects or lack of effects they are seeing has as much to do with the variable coherence/incoherence between the visual and auditory stimuli as anything.

My unease about this being a “just so” story is heightened all the more by the fact that the justification for their particular data patterns are couched in findings from their own prior research using the same probe localization and the same stimuli, and to the degree others are cited, it seems to follow a rather selective pattern. As just one example, the authors claim that infants initially exhibit greater responses to auditory non-social stimuli relative to social stimuli and cite their own in press paper in support of this. I am unfamiliar with such a pattern, but this is the basis for much of the reasoning throughout the paper. Aside from their own work, the authors cite work from two other labs but don’t go into detail about the basis for the finding. This seems like important detail to provide.

Ultimately, I appreciate the importance of the direction the work is headed in. I am not, however, comfortable with the strong claim the authors currently are making, despite their claim (in the discussion) that they “should be careful not to infer any predictive qualities of these results.” They should be careful;

they are not. The work is exploratory in nature and should be presented as such.

Specific points:

The characterization of activation as “diminished” seems odd.

The attrition rate in the fNIRS testing seems quite high (despite authors’ claims that it is standard for the field, they cite themselves in making that claim). What does this mean about the overall conclusions from this domain of research?

Is there a concern about order effects in the repeated looping of the same order of conditions? Why was this done this way?

Looking time had to be greater than 60% for a trial to be retained. Did the authors track when the looking was off the screen and how that corresponded with visual responses? No statistic is provided for what the average looking time was and 60% seems low.

p. 15: not exactly sure what is meant by this: “which one might expect from this preliminary sample size looking at a putative endophenotype”

The term “non-vocally selective response” is hard to parse and is used throughout the paper.

The paper has lots of field-specific jargon. In particular, the authors repeat the term “dimensional approach” at various points and the way it is used makes me wonder what exactly the authors mean by it (e.g., dimensional brain-behaviour relationship). Another term that gets used rather loosely is “trait-level effect”...huh? Be specific.

Figure 4 is not at all clear visually; something wrong with the formatting.

The authors make many claims based on nonsignificant results.

The discussion is *far* too long and defensive in tone.

Authors’ Response 26 June 2017

Author’s response to Reviewers’ comments:

Reviewer 1:

1) The authors are clearly aware of their study limitations (and did an excellent job in the discussion addressing them), but the title conveys a stronger message than is warranted by the preliminary nature of the findings. Similarly, trend-level results (e.g., for the visual social-nonsocial analysis) may not be sufficient to support the argument for an endophenotype.

We thank the reviewer for their praise of our Discussion. Given the potential implications of these results, caveated by the small sample size, we were careful to nuance the Discussion section accordingly. However, we agree that the wording of the original title was too strong and so have modified to the following:

“Cortical responses before six months of life associate with later autism”

2) Given the previously published work from this group using the same paradigms, it is surprising that the analyses are only retrospective (why not explore the prospective associations between the reported fNIRS measures indexing group differences at 4-6 months and the later outcomes?), and the hypotheses are not specific in regard to the ROIs. Of particular concern is that the fNIRS findings of this retrospective analysis do not appear to align well with the channels/ROIs previously identified in infant data as sensitive to risk group membership.

We have given this comment a great deal of thought. Our choice of using an ROI analysis was driven by a trend in the fNIRS field to use ROI approaches rather than individual channel analyses with the introduction of higher density NIRS channel arrays. In addition, we have published a correction of our previously reported low-high risk group differences (Lloyd-Fox et al., 2013) in

EJN European Journal of Neuroscience FENS Federation of European Neuroscience Societies

which we report an error in the time window of our analyses (Lloyd-Fox et al., 2016). With the corrected time window the difference between low and high risk groups was actually far greater than that reported in the original publication, and extended to the ROIs that were used here. These two factors led to the current presentation of results. However we can see that for the reader this may not be the best approach for comparing to the previous paper, or for assigning clear hypotheses. Therefore we have attempted to represent the data both from a perspective informed by the previous publication, and a hypothesis driven approach, which led to several changes in the Introduction, Results and Discussion (these can be found in the main manuscript highlighted in grey). An outline of these changes are detailed here:

a) *Firstly, we have used the ROI approach to assess group differences in averaged responses as it is more conservative and robust to noise in the data. Following your comment regarding the hypotheses driving the choice of ROI we have changed the ROIs selected for each contrast. Rather than use all ROIs for both contrasts, we now use previous literature to support the selection of ROIs appropriate for each contrast (visual and auditory). We have now made changes to these ROIs as follows:*
    i. *For the visual contrast we have used IFG and pSTS-TPJ – these ROIs reflect the social brain regions highlighted in previous research of visual social cues that were available within our array design in typically developing adults and infants (Pelphrey et al., 2005; Lloyd-Fox et al., 2009, 2017).*
    ii. *For the auditory contrast we have used MTG-STG and pSTG-TPJ – these ROIs cover the areas of activation seen in NIRS and MRI studies of adult and infant vocal – non-vocal responses (Belin et al., 2000, Gervais et al., 2004, Grossman et al., 2010, Minigawa-Kawai et al., 2011, Lloyd-Fox et al., 2011, 2013, 2017).*
    iii. *In addition we slightly shifted the position of the MTG-STG ROI to align with the location showing vocal>non-vocal selectivity in the low risk and HR-noASD group channel analysis (and which consistently evidenced vocal selectivity in a previously published longitudinal study over the first two years of life (Lloyd-Fox et al., in press).*
    iv. *As a consequence of shifting this MTG-STG ROI, one HR-noASD infant had missing data in the MTG ROI for the auditory contrast in one hemisphere, and so we changed the analyses to a linear mixed modelling approach to account for missing data, rather than lose the data from this participant.*
    v. *For clarification, the ROIs used in the current paper encompass the location of the channels that previously showed risk group differences in activation in the previous publication to the visual and auditory contrasts (Lloyd-Fox et al., 2013). Given that the window used in Lloyd-Fox et al., 2013 was too early (4-12s rather than 8-12s - see Lloyd-Fox et al., 2016), we ran the channel by channel analysis again for the low risk infants to double check the regions of activation in the later time window. This showed that there are a higher number of channels showing significant low risk responses in ROIs, which replicated locations found in other publications (Belin et al., 2000; Grossman et al., 2010; Lloyd-Fox et al., 2011, 2017). Therefore our ROIs encompass the regions of the social brain network and vocally selective auditory regions reflected by the corrected low risk responses reported here in Supplementary data, and the location of responses found in previous literature on typically developing infants and adults.*

b) *Secondly, we have now presented the channel by channel analysis of the peak response at 8 – 16s post stimulus onset in the supplementary data for the 3 outcome groups (low risk, HR-noASD and HR-ASD).*
    i. *This window was the intended window for peak analyses in Lloyd-Fox et al., 2013, whilst the window actually reported in the previous publication was 4-12s (see Lloyd-Fox et al., 2016).*
    ii. *The supplementary figure shows uncorrected p-values (to replicate the approach used in the 2013 paper) and the accompanying table highlights those channels which survive a multiple comparison correction.*
    iii. *There are significant responses within the 3 outcome groups in channels encompassed by the ROIs. We haven't retrospectively assigned ROIs to emphasise group differences, but rather used ROI locations generated from the risk paper and previous literature.*

iv. *Group differences were not investigated in the channel by channel analyses of peak activation*

c) *As a consequence of these changes we have restructured the final section of our Introduction to give the reader a clearer sense of our hypotheses:*

"Previous research allowed us to make the following hypotheses. Firstly, we predicted that within IFG and pSTS-TPJ social brain regions the HR–ASD infants would show reduced responses to the visual social stimuli compared with the non-social stimuli relative to the low risk (LR) infants. Secondly, given previous findings in low risk infant cohorts (Lloyd-Fox *et al.*, in press, 2013) and adults (Gervais *et al.*, 2004) we predicted that the MTG-STG region would evidence greater vocal selectivity (vocal > non-vocal responses) in the low risk (LR) infants compared with the HR-ASD infants. Thirdly, given the extensive non-vocal selectivity (non-vocal > vocal) in pSTS-TPJ regions seen in the high risk group in our previous publication (Lloyd-Fox *et al.*, 2013), we predicted that the HR-ASD infants were driving this response and would therefore show enhanced non-vocal selectivity relative to the LR groups. Finally, given that there is evidence of a broader autism phenotype within individuals with a familial risk of ASD we also hypothesized that the high-risk infants who did not go on to develop ASD (HR–noASD) would show an attenuated atypical response relative to the HR-ASD infants, while being less "typical" compared to the low risk group."

d) *We hope that our analyses are now structured more clearly and better aligned with our hypotheses. In summary our findings are: For the linear mixed modelling group contrasts, all contrasts between low risk and HR – ASD infants are significant (both for visual and auditory). The auditory contrast also shows a significant difference between HR – ASD and HR – noASD groups. The visual contrast shows a trend difference between the HR – ASD and HR – noASD groups in the right direction and as we discuss should be followed up with larger sample sizes in future cohorts. The correlational analyses between the brain responses to the visual and auditory contrasts and the behavioural symptomology of ASD are also significant. Finally, the channel by channel analyses in the Supplementary data for each group provide support for these group contrast findings.*

e) *Upon request from the Editors we how include an additional figure (Figure 4) showing individual data in more detail, rather than use the bar graphs. Furthermore, these figures of individual infant responses support the pattern of responses that we describe at the group level.*

3) Furthermore, in this manuscript, the low-risk and HR-noASD groups demonstrated no evidence of vocal-nonvocal sound discrimination (the activation difference scores near zero), which contradicts previous reports of greater sensitivity to vocal than nonvocal stimuli in 4-6-month-old LR infants. The discussion does not address these discrepancies, which brings into question the relative utility of the results from this and/or prior studies.

Whilst we agree with the reviewer that our vocal selective response was small in the previous version of the manuscript, note that the response that you saw was averaged over 3 ROIs (IFG, aMTG-STG and pSTS-TPJ) rather than within the ROI that we expected to see the vocal selective response. Now that we have removed the IFG ROI from this condition contrast in the current manuscript the vocally selective response (across aMTG-STG and pSTS-TPJ ROIs) are of greater magnitude. Furthermore, the effect sizes (Cohen's d) of the vocally selective responses in the individual channels for the low risk and HR-noASD groups ranged from 0.52-0.66. These effect sizes are consistent with those reported in previous studies (i.e. Lloyd-Fox et al., 2017 ranges from 0.4 – 1.07.

Perhaps what makes the vocal-non-vocal responses in the low risk and HR-noASD groups seem low, is that the magnitude of the non-vocal selectivity seen in the HR ASD infants is relatively so much higher than other low risk cohorts showing non-vocal selective responses. For example, the non-vocal selectivity seen in the 0-2 and 4-8 mth olds in the longitudinal Lloyd-Fox et al., 2017 study had effect sizes of 0.48 and 0.25, respectively whilst the effect size of the HR-ASD infants non-vocal selective response was 0.9.

Therefore, we believe the current results, with the newer analyses will allay your concerns and are

in line with previous research of low risk findings.

Additional comments:

4)

The introduction could be strengthened by providing a more detailed background (e.g., p.4 "we have identified regions…" – which ones? P.5 "we reported diminished fNIRS activation…" – in what specific regions?). Similarly, please clarify the typical time course of emerging speech selectivity (p.21 "a few months later" is to generic).

We thank the reviewer for these comments and have edited and added additional information into the Introduction as given here:

"Through a series of fNIRS studies with infants aged six months and under (Grossmann *et al.*, 2008; Lloyd-Fox *et al.*, 2009, 2011; Correia *et al.*, 2012; Farroni *et al.*, 2013; Grossmann *et al.*, 2013; Lloyd-Fox, *et al.*, 2014), researchers have evidenced enhanced activation to dynamic visual social stimuli (such as facial eye and mouth movements and nursery rhyme hand actions) in prefrontal, inferior frontal and superior temporal regions of the social brain network. These findings are consistent with patterns of activation found in studies with similar aged infants viewing static social and non-social stimuli (Otsuka *et al.*, 2007; Carlsson *et al.*, 2008; Nakato *et al.*, 2009) and older infants viewing dynamic social stimuli (Minagawa-Kawai *et al.*, 2009; Ichikawa *et al.*, 2010; Urakawa *et al.*, 2015; Lloyd-Fox *et al.*, 2017) and fMRI studies with adults (Allison *et al.*, 2000; Pelphrey, Morris, Michelich, *et al.*, 2005; Lotze *et al.*, 2006; Van Overwalle & Baetens, 2009). Further, auditory social responses localized over regions of the middle and superior temporal gyri and sulci (particularly anterior regions) - to vocalisations and auditory communicative cues - have also been evidenced in fNIRS studies with young infants (Grossmann *et al.*, 2010; Minagawa-Kawai *et al.*, 2011; Lloyd-Fox *et al.*, 2012; 2014; 2015), replicating and extending fMRI research with adults (Belin *et al.*, 2000). Thus, in these infant studies, regions have been identified (1) over IFG and pSTS-TPJ that show enhanced activation to *visual* dynamic social stimuli (i.e. actors playing Peek-a-boo) relative to both non-social static images (i.e. cars, trains) and dynamic videos (i.e. machinery, toys turning); and (2) over aMTG-STG that respond to *auditory* human generated stimuli (i.e. non-communicative and communicative vocalisations) to a greater degree than non-social stimuli (environmental sounds i.e. rattles, water). Further, recent research suggests that in the first months of life (0 - 4 months of age) infants exhibit non-discriminative and/or larger responses to auditory non-vocal stimuli relative to vocal stimuli (Grossmann *et al.*, 2010; Cristia *et al.*, 2014; Lloyd-Fox *et al.*, 2017) in bilateral aMTG-STG and pSTS-TPJ regions, which then diminishes with age (i.e. absent in a group of 7 month olds and in longitudinal research by approximately 9-13 months of age when studied from 4 months upwards (Grossmann *et al.*, 2010; Lloyd-Fox *et al.*, 2017)). Whilst over the same time period specialisation to auditory vocal stimuli (vocal > non-vocal sounds) in aMTG-STG increases and becomes more stable over the second half of the first year of life (5 months onwards) and into toddlerhood (Grossmann *et al.*, 2010; Lloyd-Fox *et al.*, 2012, 2017). Interestingly, an fMRI study revealed that at 1 – 4 months of age infants left temporal cortex responded to any communicative sounds, speech or communicative vocalisations (i.e. laughing, disgust) stronger than to non-communicative vocalisations (i.e. yawning, coughing) and environmental sounds (i.e. water)(Shultz *et al.*, 2014). Whilst these studies did not directly investigate the same processing (i.e. Shultz et al. did not directly investigate responses in other brain regions or non-vocal selectivity, and Grossman et al., Lloyd-Fox et al did not separate communicative from non-communicative sounds) taken together these studies support a developmental pathway of specialisation to communicative sounds and speech over the first year of life relative to non-communicative and environmental sounds."

AND

"Furthermore atypical functional lateralization to language has been found in children and adults (Kleinhans *et al.*, 2008; Redcay & Courchesne, 2008; Anderson *et al.*, 2010; Eyler *et al.*, 2012). For example, fMRI research with young children later diagnosed with ASD (Eyler *et al.*, 2012), aged 1-4 years, found deficient specialisation to language in the left hemisphere which became more pronounced in the older aged participants."

AND

"Of interest, several studies have indicated anatomical and connectivity atypicalities in temporal regions, particularly the STS, in children and adults with ASD (Boddaert *et al.*, 2004; Zilbovicius *et al.*, 2006; Zhu *et al.*, 2014). Whether these atypical visual and auditory responses are linked causally to underlying atypical anatomy from this early in life, or whether atypical early function drives anatomical differences has yet to be established. Examining the brain correlates associated with processing social stimuli at an earlier age will help us to understand the developmental trajectory of these atypicalities further and to define infant autism antecedents."

5)

Visual and auditory hemodynamic data are presented inconsistently: only difference values are presented in Fig.2 (visual) while separate conditions are plotted in Fig.3 (auditory).

Due to the way in which the stimuli were presented it is impossible to present the visual and auditory haemodynamic data in exactly the same way. The auditory manipulations are further experimental conditions contained within the visual presentation. While the visual response is derived from a difference of the visual social stimuli from the non-social baseline. We have added additional detail into the Methods section for clarification.

6)

Please clarify the rationale for pairing the auditory stimuli with social videos and not a different visual stimulus (bubbles? abstract screensavers?).

The design of our experiment is due to a trade-off between the optimal counterbalanced design, the practical considerations of limited attention span when working with infants, and the multi-paradigm nature of the longitudinal project that this NIRS session was within. We only have a limited period of attention for each infant and we chose a design that would maximise the data we collect.. While it would have been optimal to include trials with a non-social dynamic visual stimulus (accompanied by either silence, vocal, or non-vocal sounds), this would have nearly doubled the total length of our experiment and thus significantly increased data and participant loss. If we had replaced the social videos in the auditory trials with non-social videos we would then have introduced an issue in the visual comparison that we made as we would have shown the infants more visual non-social trials (in baseline periods between each experimental condition trial) than the visual only social trials (which would have been reduced to a third as many, i.e. present in 1 of the 3 experimental conditions, rather than all 3). This imbalance in trial numbers could have led to differential habituation and confounded our findings.

We acknowledge the possibility of cross-modal effects given our experimental design (and have included this as a discussion point), though we were careful to make the visual and auditory stimuli non-synchronous and pseudo-randomised. However, we do not believe that cross-modal effects with the use of the social videos rather than a non-social video are a significant contributor to our findings as the voice-selective effects in the low-risk group largely replicate those of previous fMRI and fNIRS studies in adults and infants. In these previous studies the response is evident in the temporal cortex whether the auditory stimuli are accompanied by visual stimuli (Grossman et al., 2010, LloydFox et al., 2011) or no visual stimuli (Belin et al., 2000; Blasi et al., 2011). Further the multi-modal presentation in Grossman et al. (2010) used non-human dynamic visual stimuli alongside the vocal and non-vocal auditory stimuli yet still found similar patterns of voice-selective activation to the current study (for an overview schematic of similar vocal > non-vocal responses across different stimuli combinations see Lloyd-Fox et al., 2011).

Reviewer: 2

Comments to the Author
EJN-2016-12-24223 (ASD): Cortical signature of autism evidence before six months of life

The manuscript reports behavioral and neurophysiological data collected from ~36 children at two times points: first, between 4 and 6 months and again at 36 months. Children were either low risk

or high risk for Autism Spectrum Disorder. High risk children were infant siblings of children who had been clinically diagnosed as having ASD. The authors compare neural responses collected at Time 1 using functional near infrared spectroscopy in response to social/nonsocial visual/auditory combinations of stimuli based on the diagnostic status of the children at Time 2. They interpret their findings as indicating that fNIRS can be used to identify a neural marker of ASD within the first year of life.

Please note that the analyses and figures have been changed in response to comments from Reviewer 1 and the Editors. These have been described fully in reply to Reviewer 1's point 2. Please refer to this for an explanation of our updated anaylsis pathway and findings.

(1) I have a mixed assessment of this work. On the one hand, it is important to identify early markers of ASD for targeting early intervention. On the other, these results are centered on only five children who ultimately were diagnosed with ASD. Making any claims based on an N of 5 would make me uncomfortable; making claims about a "cortical signature of autism" as the title and content of this manuscript do make me doubly so.

The authors do a good job justifying every aspect of the data that doesn't cohere and they anticipate a wide variety of counter arguments in their discussion section (so much so, it seems safe to say that this work has been rejected in various ways by various journals). The problem is that their conclusion is an easy one given the huge body of data preceding them that would point to the possibility of a neural signature for ASD.

Given the potential implications of these results, caveated by the small sample size, we were careful to nuance the Discussion section accordingly, reflecting our caution about overstating the results. However, please note that our findings do not rest solely on group comparisons, but can also be seen in the individual data presented in Figure 4 and the dimensional analyses with behavioural measures, an approach highly encouraged in the field and by the Editors. Upon reflection we agree that the original title was too strong and so have modified to the following:

"Cortical responses before six months of life associate with later autism"

For the record, we note that this manuscript has not previously received multiple rejections as the reviewer suggests. The reviewer's final sentence we find puzzling, and we are not sure how to respond to what appears to be a comment about the field in general.

(2) The problem with their data is the relatively gross localization of their probe (no digital localization was conducted) and the averaging across large chunks of cortex their data are based on.

When the infant data was collected for this study (2010-2012) digital localisation techniques were not to an adequate standard for studies of awake young infants. Our lab has closely followed the developments in this technology and carried out repeated beta testing of this technique but at the time of data collection it was still slow to use and taxed the attention span of 4 – 6 month olds, requiring them to sit through the study and then further collection of localisation data. However, we believe that the alternative methods that we followed gave us confidence about the localisation of the channels over anatomy. The co-registration work (Lloyd-Fox et al., 2015) was undertaken with careful measurements and digital photos of the arrays and infant head to localise with infant age appropriate templates. Furthermore, a third of these infants contributed individual MRI and NIRS data to the co-registration paper, so we were able to check individual datasets for the location of the ROIs. We have also carried out several comparisons of head measurements, age, Polhemus digitisation and relation to anatomy in individual MRIs with collaborators at John Richard's lab at USC (because they have a database of high quality infant age specific 3T MRI scans), to see how external landmarks relate to underlying anatomy and found consistent locations across individuals if matched for age and head measurements without it being necessary to use digitised localisation. This gave us further confidence in this co-registration method. The infant atlas and individual MRI work showed responses could be located within macro-anatomical regions comparable to the locations described in previous adult MRI work on vocal selectivity and visual social responses. In the adult MRI papers that we reviewed, while the location of maximal activation may be precise, the extent of activation was often evident over a wide area in papers within these social brain regions

(i.e. Belin et al., 2000 – "voice-sensitive activity was distributed in three bilateral clusters along the STS: one in the anterior portion close to the temporal pole; one in the central portion approximately at the level of the anterior extension of Heschl's gyrus; and one in the STS portion posterior to Heschl's gyrus, extending dorsally and caudally to the planum temporale of the superior temporal gyrus" Belin et al., (2000), Nature, 403, pg 309. These clusters included middle and superior temporal gyrus and sulci, and were present in all individual participants in some or all of these regions). Therefore we were keen to maintain a fairly wide ROI to account for variance in the location in previous adult findings and to account for individual variability in the location of the maximum response.

(3) The brain regions in question here are relevant to all sorts of processing. If one didn't have the prior knowledge of what the ASD data should show that would follow a clear story, the data could very well be accounted for in a variety of other ways. The primary alternative explanation I kept returning to was that whatever effects or lack of effects they are seeing has as much to do with the variable coherence/incoherence between the visual and auditory stimuli as anything.

We agree with the reviewer that these brain regions are recruited for other types of processing in addition to social processing and had acknowledged alternative potential explanations in the Discussion section including the temporal aspects and high fidelity of processing of dynamic stimuli and cross modal effects of visual/auditory presentation. We have now adapted our Discussion further to address your concerns, added additional text concerning the processing of multi-sensory presentation of stimuli and nuanced our concluding sentence to contain the following:

"Future research should further explore the origins of these atypical brain responses, how these findings may relate to the complexity and multisensory nature of the social vs. non-social stimuli used, how they relate to genetic and other neural mechanisms, and how they may in turn affect more complex social learning and social interaction throughout later development. "

And additional text in Discussion:

"With regard to the auditory effects, as we acknowledged previously (Lloyd-Fox *et al.*, 2013), there is a possibility that infants at-risk for ASD process multisensory stimuli differently from their typically developing counterparts, due to the incongruent presentation of visual and auditory stimuli. Though we were careful to ensure the visual and auditory stimuli were non-synchronous and pseudo-randomised, our design was clearly restricted by what is possible with infants in a limited time period. We do not believe that cross-modal effects are a significant contributor as the low risk findings largely replicate those of previous fMRI and fNIRS studies in adults and infants, whether or not the auditory stimuli were accompanied by visual stimuli (Belin *et al.*, 2000; Grossmann *et al.*, 2010; Blasi *et al.*, 2011; Lloyd-Fox *et al.*, 2012). Indeed the multi-modal presentation in Grossman et al. (Grossmann *et al.*, 2010) used non-human dynamic visual stimuli alongside the vocal and non-vocal auditory stimuli yet still found similar patterns of voice-selective activation to the current study using social dynamic visual stimuli. However, given recent evidence suggesting that individuals with ASD may have atypical multisensory processing, for example the presence of speech has been shown to disrupt processing of videos of complex social stimuli (Shic *et al.*, 2014), in future work we aim to disentangle how different components of the current stimuli may have contributed to the atypical response seen here."

(4) My unease about this being a "just so" story is heightened all the more by the fact that the justification for their particular data patterns are couched in findings from their own prior research using the same probe localization and the same stimuli, and to the degree others are cited, it seems to follow a rather selective pattern. As just one example, the authors claim that infants initially exhibit greater responses to auditory non-social stimuli relative to social stimuli and cite their own in press paper in support of this. I am unfamiliar with such a pattern, but this is the basis for much of the reasoning throughout the paper. Aside from their own work, the authors cite work from two other labs but don't go into detail about the basis for the finding. This seems like important detail to provide.

Whilst we acknowledge that the research that forms the basis of some of our hypotheses originate from our own lab, which is partly driven by the limitation in the number of fNIRS and fMRI infant

EJN European Journal of Neuroscience FENS Federation of European Neuroscience Societies

studies published in this field, we believe that there is also considerable research with adults and older infants to support the hypotheses and regret that this did not come through in our previous version of the paper. We have now revised several sections of the paper to address your concerns. Furthermore, in light of this comment and a comment from Reviewer 1 we have given more detailed hypotheses and included additional supporting literature into the Introduction and provided more detail about other research findings.

"Through a series of fNIRS studies with infants aged six months and under (Grossmann *et al.*, 2008; Lloyd-Fox *et al.*, 2009, 2011; Correia *et al.*, 2012; Farroni *et al.*, 2013; Grossmann *et al.*, 2013; Lloyd-Fox, *et al.*, 2014), researchers have evidenced enhanced activation to dynamic visual social stimuli (such as facial eye and mouth movements and nursery rhyme hand actions) in prefrontal, inferior frontal and superior temporal regions of the social brain network. These findings are consistent with patterns of activation found in studies with similar aged infants viewing static social and non-social stimuli (Otsuka *et al.*, 2007; Carlsson *et al.*, 2008; Nakato *et al.*, 2009) and older infants viewing dynamic social stimuli (Minagawa-Kawai *et al.*, 2009; Ichikawa *et al.*, 2010; Urakawa *et al.*, 2015; Lloyd-Fox *et al.*, 2017) and fMRI studies with adults (Allison *et al.*, 2000; Pelphrey, Morris, Michelich, *et al.*, 2005; Lotze *et al.*, 2006; Van Overwalle & Baetens, 2009). Further, auditory social responses localized over regions of the middle and superior temporal gyri and sulci (particularly anterior regions) - to vocalisations and auditory communicative cues - have also been evidenced in fNIRS studies with young infants (Grossmann *et al.*, 2010; Minagawa-Kawai *et al.*, 2011; Lloyd-Fox *et al.*, 2012; 2014; 2015), replicating and extending fMRI research with adults (Belin *et al.*, 2000). Thus, in these infant studies, regions have been identified (1) over IFG and pSTS-TPJ that show enhanced activation to *visual* dynamic social stimuli (i.e. actors playing Peek-a-boo) relative to both non-social static images (i.e. cars, trains) and dynamic videos (i.e. machinery, toys turning); and (2) over aMTG-STG that respond to *auditory* human generated stimuli (i.e. non-communicative and communicative vocalisations) to a greater degree than non-social stimuli (environmental sounds i.e. rattles, water). Further, recent research suggests that in the first months of life (0 - 4 months of age) infants exhibit non-discriminative and/or larger responses to auditory non-vocal stimuli relative to vocal stimuli (Grossmann *et al.*, 2010; Cristia *et al.*, 2014; Lloyd-Fox *et al.*, 2017) in bilateral aMTG-STG and pSTS-TPJ regions, which then diminishes with age (i.e. absent in a group of 7 month olds and in longitudinal research by approximately 9-13 months of age when studied from 4 months upwards (Grossmann *et al.*, 2010; Lloyd-Fox *et al.*, 2017)). Whilst over the same time period specialisation to auditory vocal stimuli (vocal > non-vocal sounds) in aMTG-STG increases and becomes more stable over the second half of the first year of life (5 months onwards) and into toddlerhood (Grossmann *et al.*, 2010; Lloyd-Fox *et al.*, 2012, 2017). Interestingly, an fMRI study revealed that at 1 – 4 months of age infants left temporal cortex responded to any communicative sounds, speech or communicative vocalisations (i.e. laughing, disgust) stronger than to non-communicative vocalisations (i.e. yawning, coughing) and environmental sounds (i.e. water)(Shultz *et al.*, 2014). Whilst these studies did not directly investigate the same processing (i.e. Shultz et al. did not directly investigate responses in other brain regions or non-vocal selectivity, and Grossman et al., Lloyd-Fox et al did not separate communicative from non-communicative sounds) taken together these studies support a developmental pathway of specialisation to communicative sounds and speech over the first year of life relative to non-communicative and environmental sounds."

AND

"Furthermore atypical functional lateralization to language has been found in children and adults (Kleinhans *et al.*, 2008; Redcay & Courchesne, 2008; Anderson *et al.*, 2010; Eyler *et al.*, 2012). For example, fMRI research with young children later diagnosed with ASD (Eyler *et al.*, 2012), aged 1-4 years, found deficient specialisation to language in the left hemisphere which became more pronounced in the older aged participants."

AND

"Of interest, several studies have indicated anatomical and connectivity atypicalities in temporal regions, particularly the STS, in children and adults with ASD (Boddaert *et al.*, 2004; Zilbovicius *et al.*, 2006; Zhu *et al.*, 2014). Whether these atypical visual and auditory responses are linked causally to underlying atypical anatomy from this early in life, or whether atypical early function drives anatomical differences has yet to be established. Examining the brain correlates associated

with processing social stimuli at an earlier age will help us to understand the developmental trajectory of these atypicalities further and to define infant autism antecedents."

(5) Ultimately, I appreciate the importance of the direction the work is headed in. I am not, however, comfortable with the strong claim the authors currently are making, despite their claim (in the discussion) that they "should be careful not to infer any predictive qualities of these results." They should be careful; they are not. The work is exploratory in nature and should be presented as such.

We hope that changes that we have made in the Title, Abstract, Introduction and Discussion to highlight the preliminary nature of the findings are now clearer.

Specific points:

(6) The characterization of activation as "diminished" seems odd.
We have replaced this word with "reduced" or "atypical" as and when appropriate.

(7) The attrition rate in the fNIRS testing seems quite high (despite authors' claims that it is standard for the field, they cite themselves in making that claim). What does this mean about the overall conclusions from this domain of research?

The citation that we refer to was from a review which contained a meta-analysis of all infant fNIRS studies published up to the date that the review was published. This is why we referred to this paper as it gave a good overview of the field. However, to address this criticism we have added a further two reviews which were written in collaboration across several research labs using fNIRS and overviewed the published fNIRS infant studies at more recent dates. The findings from these papers report similar ranges of attrition.
With regard to the current study, we realised that we had mistakenly added an incorrect value for the attrition rate (it was actually the number of infants included in the study) and so have corrected this, many thanks for bringing this to our attention. Further, after consideration of your comment we have removed 5 infants from this calculation (and total number tested) as these infants did not actually contribute any data due to unforeseen equipment failure occurring and so it would be fairer to remove these from this calculation. Finally, we have added a sentence into this section to give readers a sense of why this attrition rate may still be fairly high,

"The fNIRS data presented in the current study originates from the first visit that the infants attended as part of the prospective long-term project at the Centre for Brain and Cognitive Development. A further 24 infants (60% inclusion rate) participated but were excluded from the study (17 high-risk infants, 7 low-risk infants), details of which are found in the Data Processing section of the Methods. Note that 37 high-risk and 23 low-risk infants took part in this study, hence the disparity in numbers between groups. "

AND

"Consequently, of the 60 infants tested 24 were excluded (60% included in sample); 18 - due to an insufficient number of valid trials according to looking time measures, 5 – due to a high level of rejected fNIRS data (artifact detection algorithms and analyses) and 1 due to missing the 36 month clinical assessment as the family moved abroad. Note that while this attrition rate was fairly high - likely a consequence of our 3-condition design requiring infants' to view a high number of trials - it did not differ between the low and high risk infants and was within the typical range for infant fNIRS studies (Lloyd-Fox *et al.*, 2010; Gervain *et al.*, 2011; Cristia *et al.*, 2013)."

(8) Is there a concern about order effects in the repeated looping of the same order of conditions? Why was this done this way?

We have extended this section of the Methods to include the full loop of trials used "V-S, A-NV, A-V, V-S, A-V, A-NV, V-S, A-V, A-NV, V-S, A-NV, A-V". *As can be seen, this is a complex repeat pattern and so we hoped it would not be predictive. We chose to present the trials in this pseudo random order as we wished to ensure that infants viewed an equivalent number of each condition after a*

certain amount of time.

(9) Looking time had to be greater than 60% for a trial to be retained. Did the authors track when the looking was off the screen and how that corresponded with visual responses? No statistic is provided for what the average looking time was and 60% seems low.

As stated at the beginning of the Results section, information about looking time and number of presented and valid trials was given in the Supplementary Information in Table S4. If preferred, we can move this to the main paper?
The average looking time % per trial for each group was the following: Low risk (94.18%: SD 3.2), HR no ASD (92.59%: SD 5.76), HR ASD (91.05%: SD 2.4). There were no significant differences across groups.

(10) p. 15: not exactly sure what is meant by this: "which one might expect from this preliminary sample size looking at a putative endophenotype"
We have removed this sentence, as agree that it is unnecessary here.

(11) The term "non-vocally selective response" is hard to parse and is used throughout the paper.

If the reviewer has a suggestion for an alternative term we are happy to consider changing this phrase. Given that the original fMRI studies with adults referred to responses as voice- or vocal-selective, we, and others, chose to apply the same terminology in our infant studies to the non-vocal responses to match the vocal contrast (Belin et al., 2000; Gervais et al., 2004; Grossman et al., 2010; Lloyd-Fox et al., 2011, 2013, 2014, 2016)

(12) The paper has lots of field-specific jargon. In particular, the authors repeat the term "dimensional approach" at various points and the way it is used makes me wonder what exactly the authors mean by it (e.g., dimensional brain-behaviour relationship). Another term that gets used rather loosely is "trait-level effect"…huh? Be specific.
To help the reader we have defined the term dimensional approach when first used:
"A dimensional approach (relations of quantitative scores in neuroimaging and behavioural measures)…"
We also removed "trait-level" and replaced with the following (on page 17):
"To further investigate whether these findings reflect a population level effect (driven by traits (characteristics) evident across individuals) or are driven by an effect of HR-ASD outcome…"
and here (on page 19):
"In the current study, the reduced response to visual social stimuli was also found to be associated with increased traits of atypical social responsiveness (characteristics linked to ASD) across all infants."

(13) Figure 4 is not at all clear visually; something wrong with the formatting.
Thanks for bringing this to our attention, we will speak to the editorial team and try and rectify the problem as visually it looks fine in the version that was approved upon submission.

(14) The authors make many claims based on nonsignificant results.
We hope that the new analyses provide further support for our claims. For the linear mixed modelling group contrasts, all contrasts between low risk and HR – ASD infants are significant (both for visual and auditory). The auditory contrast also shows a significant difference between HR – ASD and HR – noASD groups. While the visual contrast shows a trend between the HR – ASD and HR – noASD groups the trend is in the right direction and as we discuss should be followed up with larger sample sizes in future cohorts. The correlational analyses between the brain responses to the visual and auditory contrasts and the behavioural symptomology of ASD are also significant. Finally, the channel by channel analyses requested by Reviewer 1 for each group provide support for these group contrast findings.

(15) The discussion is *far* too long and defensive in tone.

As described in reply to previous comments, we hope that the Discussion is now appropriate and addresses your concerns. We have also thoroughly reviewed the text and cut sections to reduce the overall length.

EJN European Journal of Neuroscience FENS Federation of European Neuroscience Societies

2nd Editorial Decision 29 August 2017

Dear Dr. Lloyd-Fox,

Your resubmitted manuscript has been reviewed by the external reviewers as well as by the Section Editor, Dr. Sophie Molholm, and ourselves.

We are pleased to say you that your manuscript is considered to be much improved in this revised version. We expect that it will be acceptable for publication in EJN following the minor revisions in response to Reviewer 2. In addition please supply editable versions of the figure files and it needs to be explicitly stated that parental consent obtained to publish the image of the infant in fig 1.

If you are able to respond fully to the points raised, we would be pleased to receive a revision of your paper within 30 days.

Thank you for submitting your work to EJN.

Kind regards,

Paul Bolam & John Foxe
co-Editors in Chief, EJN

Reviews:

Reviewer: 1 (Sasha Key, Vanderbilt Kennedy Center, USA)

Comments to the Author
The revised manuscript addressed my earlier concerns.

Reviewer: 2 (Heather Bortfeld, University of California- Merced, USA)

Comments to the Author
Cortical responses before six months of life associate with later autism

This is a revision, so I will dispense with the overview of the work reported. The authors have, for the most part, been responsive to the reviews and the paper reads much more smoothly than the original version.

My primary comment focuses on the changes made in the time windows used in the analyses. I understand that there are different windows that may or may not show the best/maximum blood flow response. That said, it does seem a bit strange to move time windows on the same data from one publication to another. The authors refer to the initial window that was used (4 to 12 seconds) as an “analysis error.” But that time window doesn't reflect just the “initiation” of the cortical response, as stated on p. 14. Rather, that window includes both the build up response and the maximum response, which that no doubt pulled down the overall maximum change calculated. It seems that the authors should be clear about this. The current explanation reads as disingenuous.

This brings me to my second comment, which has to do with the HHb analyses/figures. There has been some debate in the literature about whether it is legitimate to focus solely on HbO when using fNIRS. My understanding is that one should see a robust and opposite effect for HHb to consider data for a trial/channel useable. The reality in practice is that this is not the standard to which researchers hold themselves. In the current study, the authors do include analyses of HHb, which is refreshing, but it is secondary to the focus on the HbO response (see p. 16 of the revised manuscript). The issue of the atypicality of the observed responses is mentioned in the discussion (p. 24 of the revised manuscript). Although it is good to see the authors acknowledge this, it leaves the issue muddy since the expected typical response is never discussed at the outset of the analyses. If this were a specialty

journal for the optical subdomain, it would not be so important, but this is a general interest neuroscience journal. The authors should address whether a response is considered legitimate if there is no corresponding decrease in HHb when an increase in HbO is observed (or at least acknowledge that this is a topic of active inquiry). A detailed examination of the issue may be beyond the scope of this paper, but it is important to be clear about the patterns expected/observed. While the HHb response is noted as present/not present, but the directionality of the effect isn't discussed/described. In short, the authors could make it clear at the outset of their reporting of the results what a "normal" response looks like so that the abnormal/atypical responses are understood as such.

Regarding my comment about the discussion in the original critique, I was referring indirectly to its overly lengthy and defensive tone. I referred to this directly at the conclusion of that review, assuming the authors would make the connection. Apparently they did not. In short, the statement does not reflect my view of the field, but rather the quality of the discussion in their original submission. It is much improved in this revision.

I do think Table S4 should be in the primary text, but will defer to editorial decisions on that.

Authors' Response 13 September 2017

Author's response to Reviewers' comments

Reviewer 2:

1) My primary comment focuses on the changes made in the time windows used in the analyses. I understand that there are different windows that may or may not show the best/maximum blood flow response. That said, it does seem a bit strange to move time windows on the same data from one publication to another. The authors refer to the initial window that was used (4 to 12 seconds) as an "analysis error." But that time window doesn't reflect just the "initiation" of the cortical response, as stated on p. 14. Rather, that window includes both the build up response and the maximum response, which that no doubt pulled down the overall maximum change calculated. It seems that the authors should be clear about this. The current explanation reads as disingenuous.

We agree that changing the analyses between the risk publication and outcome data publication has some drawbacks, however we believe it is important to investigate the outcome data with the updated analysis approach. This motivation is led partially by changes in analysis approaches used in fNIRS infancy research and partially by our interest in investigating whether there were latency differences in the haemodynamic responses across groups. While we did not find such differences overall, we think it was important to assess these differences in the windows chosen as the time courses indicated that the HR-ASD infants may have been taking longer to initiate and therefore reach a maximum response. In future research with larger samples we hope to assess this in more detail and so wished this paper to bridge the gap between the previous publication and future analysis approaches.

Furthermore, many thanks for highlighting concerns over the wording of this section of the Methods. We have reviewed the text and adapted to address your feedback:

"Firstly, due to an analysis error, the window (4 – 12 s) used in the previous publication was earlier than intended (as explained in an errata - Lloyd-Fox *et al.*, 2016) and so included the initiation of the haemodynamic response rather than being centred over the maximum. For a stimulus trial with this content, and of this length (9 – 12s), previous research indicates that one would expect to see the peak beyond, or towards the end of, the condition of interest (i.e. during an 8 – 16s window). Secondly, recent research analyzing the latency of the response over a large sample with a wide developmental age range from 0 – 2 years has indicated that the use of narrower windows around the peak elicits a more robust and informative marker of developmental specialisation of the haemodynamic response to this stimuli (Lloyd-Fox *et al.*, 2017). Therefore we included two narrower windows in the current analyses: the first (8 – 12s) was framed over a window likely to include the maximal responses used in the previous analyses (in Lloyd-Fox *et al.*, 2013), and the second (12 – 16s) extended beyond the end of the trial to include the remaining period of maximum activation missed in the previous publication."

EJN European Journal of Neuroscience FENS Federation of European Neuroscience Societies

2) This brings me to my second comment, which has to do with the HHb analyses/figures. There has been some debate in the literature about whether it is legitimate to focus solely on HbO when using fNIRS. My understanding is that one should see a robust and opposite effect for HHb to consider data for a trial/channel useable. The reality in practice is that this is not the standard to which researchers hold themselves. In the current study, the authors do include analyses of HHb, which is refreshing, but it is secondary to the focus on the HbO response (see p. 16 of the revised manuscript). The issue of the atypicality of the observed responses is mentioned in the discussion (p. 24 of the revised manuscript). Although it is good to see the authors acknowledge this, it leaves the issue muddy since the expected typical response is never discussed at the outset of the analyses. If this were a specialty journal for the optical subdomain, it would not be so important, but this is a general interest neuroscience journal. The authors should address whether a response is considered legitimate if there is no corresponding decrease in HHb when an increase in HbO is observed (or at least acknowledge that this is a topic of active inquiry). A detailed examination of the issue may be beyond the scope of this paper, but it is important to be clear about the patterns expected/observed. While the HHb response is noted as present/not present, but the directionality of the effect isn't discussed/described. In short, the authors could make it clear at the outset of their reporting of the results what a "normal" response looks like so that the abnormal/atypical responses are understood as such.

We are very grateful for the reviewer bringing this to our attention, indeed this advice is usually something we find ourselves recommending to other researchers, and see that we have overlooked including text on this issue in any detail earlier in the manuscript. We have now written an additional paragraph in the Methods section to address this omission:

"Either a significant increase in HbO_2, or a significant decrease in HHb, is commonly accepted as an indicator of cortical activation in infant work. If HbO_2 and HHb were either to increase or decrease significantly in unison, the signal is considered inconsistent with a haemodynamic response to functional activation (Obrig & Villringer, 2003). There was no evidence of regional simultaneous increases/decreases in HbO_2 and HHb responses in the current dataset. While many infant fNIRS studies report significant HbO_2 responses, far fewer report HHb responses, sometimes through choice, but often because they do not find significant responses (likely due to the magnitude of change in HHb generally being far lower) (Lloyd-Fox *et al.*, 2010). In accordance with previous research (Lloyd-Fox *et al.*, 2010; Gervain *et al.*, 2011; Cristia *et al.*, 2013) we found that the majority of the significant effects were in HbO_2 and so focused our results on this signal. However, we feel that it is important to report HHb responses to contribute to the field's continued effort to further our understanding of the infant haemodynamic response (see Supplementary Data for HHb responses)."

Furthermore we have clarified the direction of the significant effects in the Supplementary table of HbO_2 and HHb channel by channel responses:

"**Table S3:** Channel by channel significant increases in HbO_2 / decreases in HHb concentration during the Visual and Auditory contrasts for the LR, HR – noASD and HR – ASD participants."

3) I do think Table S4 should be in the primary text, but will defer to editorial decisions on that.

We presume the Reviewer is referring to Table S3 as there are only 3 supplementary items. Please could you clarify this to the Editors so that they can make a decision on whether to include in the main paper. We are happy to include any, or all, of the supplementary files in the main paper.

3rd Editorial Decision 28 September 2017

Dear Dr. Lloyd-Fox,

We are pleased to inform you that, pending your adding Table S2 to the main text as requested by reviewer 2, your manuscript has been accepted for publication in the special issue on autism in EJN.

If you are able to respond fully to the points raised, we shall be pleased to receive a revision of your paper within 30 days.

EJN European Journal of Neuroscience FENS Federation of European Neuroscience Societies

Thank you for submitting your work to EJN.

Kind regards,

John Foxe & Paul Bolam
co-Editors in Chief, EJN

Reviews:

Reviewer: 2 (Heather Bortfeld, University of California- Merced, USA)

Comments to the Author
The authors have responded to my concerns in sufficient detail. I meant Table S2 should be included in the main text, so that readers who are not inclined to go to supplementary material (most, in my experience) will have ready access to these important details. Many researchers discount infant looking research at least in part because these kinds of details are not provided. It is critical that such information by made readily available to the careful reader.

Authors' Response 05 October 2017

Supplementary Table S3 has been moved to the main text as Table 2.
